# Characterization of developmental and molecular factors underlying release heterogeneity at *Drosophila* synapses

**DOI:** 10.7554/eLife.38268

**Published:** 2018-07-10

**Authors:** Yulia Akbergenova, Karen L Cunningham, Yao V Zhang, Shirley Weiss, J Troy Littleton

**Affiliations:** 1 The Picower Institute for Learning and MemoryMassachusetts Institute of TechnologyCambridgeUnited States; 2 Department of BiologyMassachusetts Institute of TechnologyCambridgeUnited States; 3 Department of Brain and Cognitive SciencesMassachusetts Institute of TechnologyCambridgeUnited States; Brandeis UniversityUnited States; Baylor College of MedicineUnited States

**Keywords:** active zone, synapse, calcium channels, synaptic plasticity, synapse development, glutamate receptors, *D. melanogaster*

## Abstract

Neurons communicate through neurotransmitter release at specialized synaptic regions known as active zones (AZs). Using biosensors to visualize single synaptic vesicle fusion events at *Drosophila* neuromuscular junctions, we analyzed the developmental and molecular determinants of release probability (*P_r_*) for a defined connection with ~300 AZs. *P_r_* was heterogeneous but represented a stable feature of each AZ. *P_r_* remained stable during high frequency stimulation and retained heterogeneity in mutants lacking the Ca^2+^ sensor Synaptotagmin 1. *P_r_* correlated with both presynaptic Ca^2+^ channel abundance and Ca^2+^ influx at individual release sites. *P_r_* heterogeneity also correlated with glutamate receptor abundance, with high *P_r_* connections developing receptor subtype segregation. Intravital imaging throughout development revealed that AZs acquire high *P_r_* during a multi-day maturation period, with *P_r_* heterogeneity largely reflecting AZ age. The rate of synapse maturation was activity-dependent, as both increases and decreases in neuronal activity modulated glutamate receptor field size and segregation.

## eLife digest

To send a message to its neighbor, a neuron releases chemicals called neurotransmitters into the gap – or synapse – between them. The neurotransmitter molecules bind to proteins on the receiver neuron called receptors. But what causes the sender neuron to release neurotransmitter in the first place? The process starts when an electrical impulse called an action potential arrives at the sender cell. Its arrival causes channels in the membrane of the sender neuron to open, so that calcium ions flood into the cell. The calcium ions interact with packages of neurotransmitter molecules, known as synaptic vesicles. This causes some of the vesicles to empty their contents into the synapse.

But this process is not particularly reliable. Only a small fraction of action potentials cause vesicles to fuse with the synaptic membrane. How likely this is to occur varies greatly between neurons, and even between synapses formed by the same neuron. Synapses that are likely to release neurotransmitter are said to be strong. They are good at passing messages from the sender neuron to the receiver. Synapses with a low probability of release are said to be weak. But what exactly differs between strong and weak synapses?

Akbergenova et al. studied synapses between motor neurons and muscle cells in the fruit fly *Drosophila*. Each motor neuron forms several hundred synapses. Some of these synapses are 50 times more likely to release neurotransmitter than others. Using calcium imaging and genetics, Akbergenova et al. showed that sender cells at strong synapses have more calcium channels than sender cells at weak synapses. The subtypes and arrangement of receptor proteins also differ between the receiver neurons of strong versus weak synapses. Finally, studies in larvae revealed that newly formed synapses all start out weak and then gradually become stronger. How fast this strengthening occurs depends on how active the neuron at the synapse is.

This study has shown, in unprecedented detail, key molecular factors that make some fruit fly synapses more likely to release neurotransmitter than others. Many proteins at synapses of mammals resemble those at fruit fly synapses. This means that similar factors may also explain differences in synaptic strength in the mammalian brain. Changes in the strength of synapses underlie the ability to learn. Furthermore, many neurological and psychiatric disorders result from disruption of synapses. Understanding the molecular basis of synapses will thus provide clues to the origins of certain brain diseases.

## Introduction

Synaptic vesicle fusion occurs at specialized regions of the presynaptic membrane known as active zones (AZs). Several evolutionarily conserved structural proteins are enriched in this subdomain of the presynaptic terminal, including RIM, RIM binding protein, Syd-1, Liprin-α, ELKS/CAST/Bruchpilot, Munc13, and Bassoon/Piccolo/Fife (Schoch and Gundelfinger, 2006; Südhof, 2012; Van Vactor and Sigrist, 2017; Zhai and Bellen, 2004). These large macromolecular complexes facilitate clustering of synaptic vesicles and voltage-gated Ca^2+^ channels (VGCCs), allowing action potential-triggered Ca^2+^ influx to act locally on synaptic vesicles that are docked and primed for release (Acuna et al., 2016; Zito et al., 1999; Bucurenciu et al., 2008; Eggermann et al., 2011; Fouquet et al., 2009; Kawasaki et al., 2004). Synaptic vesicle fusion occurs through a highly probabilistic process, often with only a small percent of action potentials triggering release from individual AZs (Körber and Kuner, 2016). Although AZs largely share the same complement of proteins, release probability (*P_r_*) for synaptic vesicle fusion is highly variable across different neurons and between AZs formed by the same neuron (Atwood and Karunanithi, 2002; Branco and Staras, 2009; Melom et al., 2013; Peled and Isacoff, 2011). Studies have demonstrated that Ca^2+^ channel abundance and Ca^2+^ influx are key determinants of *P_r_* (Borst and Sakmann, 1996; Chen et al., 2015; [Bibr bib72][Bibr bib124]; Nakamura et al., 2015; Sheng et al., 2012; Wang et al., 2008). In addition, some AZ-specific proteins are non-uniformly distributed, and the molecular composition of AZs can undergo rapid changes (Glebov et al., 2017; Graf et al., 2009; Liu et al., 2016; Reddy-Alla et al., 2017; Sugie et al., 2015; Tang et al., 2016; Weyhersmüller et al., 2011; Wojtowicz et al., 1994).

The *Drosophila* neuromuscular junction (NMJ) has emerged as a useful system to study release heterogeneity. At this connection, motor neurons form glutamatergic synapses onto bodywall muscles in a stereotypical fashion, with the axon expanding to form ~10–60 synaptic boutons that each contain many individual AZs (Harris and Littleton, 2015). *Drosophila* AZs contain a similar assortment of proteins to those identified at mammalian AZs (Böhme et al., 2016; Bruckner et al., 2017, 2012; Ehmann et al., 2014; Feeney et al., 1998; Fouquet et al., 2009; Graf et al., 2012; Jan and Jan, 1976; Kaufmann et al., 2002; Kittel et al., 2006; Liu et al., 2011; Owald et al., 2010; Wagh et al., 2006). Each AZ is specifically associated with a postsynaptic glutamate receptor field. Glutamate receptors at the *Drosophila* NMJ are excitatory inotropic non-NMDA receptors that exist as tetramers, with three obligatory subunits encoded by GluRIII, GluRIID and GluRIIE, and a variable fourth subunit encoded by either GluRIIA (A-type) or GluRIIB (B-type) (Featherstone et al., 2005; Marrus et al., 2004; Petersen et al., 1997; Qin et al., 2005; Schuster et al., 1991). GluRIIA containing receptors generate a larger quantal size and display slower receptor desensitization than their GluRIIB counterparts (DiAntonio et al., 1999). The A- and B-subtypes compete for incorporation into the tetramer at individual postsynaptic densities (PSDs) in a developmental and activity-regulated fashion (Chen and Featherstone, 2005; DiAntonio et al., 1999; Marrus and DiAntonio, 2004a; Rasse et al., 2005; Schmid et al., 2008).

The stereotypical alignment of individual AZs to distinct postsynaptic glutamate receptor fields in *Drosophila* allowed the generation of genetic tools to optically follow quantal fusion events at single release sites by visualizing glutamate receptor activation (Melom et al., 2013; Peled and Isacoff, 2011). Classically, studies of synaptic transmission have used electrophysiology to measure the postsynaptic effect of neurotransmitter release over a population of release sites (Katz and Miledi, 1969, 1967), precluding an analysis of how individual AZs contribute to the evoked response. By transgenically expressing GCaMP Ca^2+^ sensors that target to the postsynaptic membrane, single vesicle fusion events at each individual AZ can be imaged by following spatially localized Ca^2+^ influx induced upon glutamate receptor opening. This allows for the generation of *P_r_* maps for both evoked and spontaneous fusion for all AZs (Cho et al., 2015; Melom et al., 2013; Muhammad et al., 2015; Newman et al., 2017; Peled et al., 2014; Peled and Isacoff, 2011; Reddy-Alla et al., 2017). One surprising observation using this quantal imaging approach is that AZs formed by a single motor neuron have a heterogeneous distribution of *P_r_*, ranging from 0.01 to ~0.5, with neighboring AZs often showing ~50-fold differences in *P_r_* (Melom et al., 2013; Peled et al., 2014; Peled and Isacoff, 2011). These differences in *P_r_* result in AZs with distinct short-term plasticity properties, suggesting release heterogeneity has functional importance for synaptic transmission (Peled and Isacoff, 2011).

Key questions raised by these observations include how *P_r_* is uniquely set for individual AZs and how the heterogeneity in *P_r_* arises during development. *P_r_* variability is likely to be controlled in part by variable Ca^2+^ channel abundance at release sites, consistent with the heterogeneity in VGCCs and other associated AZ proteins previously documented (Böhme et al., 2016; Ehmann et al., 2014; Fulterer et al., 2018; Graf et al., 2012, 2009; Guerrero et al., 2005; Peled and Isacoff, 2011). Indeed, *P_r_* has been shown to correlate with BRP abundance at AZs in *Drosophila* (Paul et al., 2015; Peled et al., 2014; Reddy-Alla et al., 2017) and BRP has a key function in clustering VGCCs (Kittel et al., 2006). Furthermore, *P_r_* has previously been shown to correlate with the number of VGCCs at several vertebrate synapses (Chen et al., 2015; Nakamura et al., 2015; Sheng et al., 2012). *P_r_* could also be regulated by local synaptic vesicle pools and their number and/or state (i.e. phosphorylation status). Beyond the molecular factors that determine AZ *P_r_*, it is unclear how release heterogeneity at *Drosophila* NMJs arises during development. Intravital imaging at third instar larval NMJs has demonstrated that AZs are born small and gain pre- and postsynaptic components over time in a sequential manner (Andlauer and Sigrist, 2012; Fouquet et al., 2009; Füger et al., 2007; Rasse et al., 2005; Zhang et al., 2010). However, this approach has not been used during earlier stages of larval development to determine whether the release heterogeneity observed at third instar NMJs reflects AZ birth order. To characterize factors regulating *P_r_* at individual AZs, as well as the origin of *P_r_* diversity, we employed optical quantal analysis and intravital imaging to examine how *P_r_* heterogeneity arises during development.

## Results

### *Drosophila* NMJ synapses display heterogeneity in *P_r_*, ranging from functionally silent sites to high *P_r_* AZs

Recent studies indicate that release sites possess structural and functional heterogeneity (Éltes et al., 2017; Holderith et al., 2012; Maschi and Klyachko, 2017; Melom et al., 2013; Peled et al., 2014; Peled and Isacoff, 2011; Reddy-Alla et al., 2017; Sugie et al., 2015). Using the *Drosophila* NMJ, we previously observed that evoked *P_r_* is non-uniform across a population of ~300 AZs formed by motor neuron MN4-Ib onto muscle 4, ranging from 0.01 to ~0.5 in HL3 saline containing 1.3 mM extracellular Ca^2+^ and 20 mM Mg^2+^ (Melom et al., 2013). In our original study, each AZ was identified by the location of postsynaptic Ca^2+^ flashes, but AZs were not directly labeled in the live preparation. To more precisely map AZ *P_r_* heterogeneity, we identified the position of each corresponding PSD by co-expressing the RFP-tagged glutamate receptor subunit GluRIIA under the control of its endogenous promoter (Rasse et al., 2005) along with a newer version of our previous biosensor, N-terminal myristoylated GCaMP6s, expressed in muscles using Mef2-GAL4. We monitored postsynaptic Ca^2+^ influx from activation of glutamate receptors after either spontaneous release or nerve stimulation (0.3 Hz for 5 min) in muscle 4 of early stage third instar larvae (Video 1).

**Video 1.** Representative movie showing evoked and spontaneous GCaMP6s events (green) in larvae expressing GluRIIA-RFP (red) that were stimulated at 0.3 Hz.

**Figure 1.**
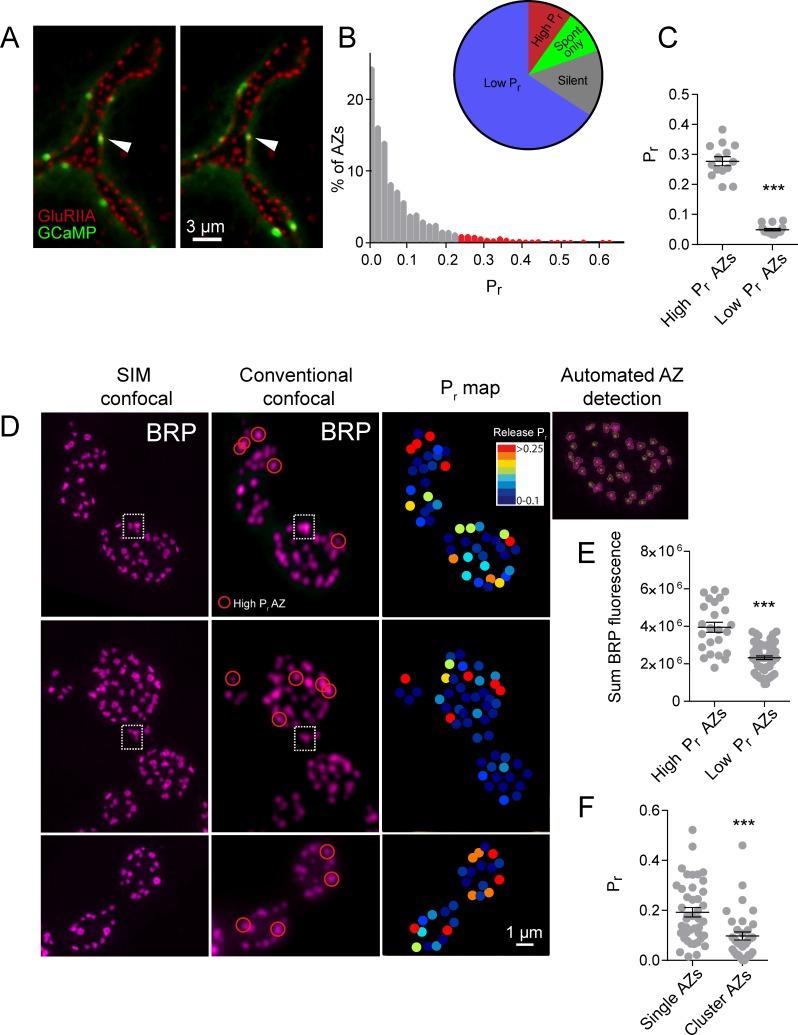
High *P_r_* sites correspond to single AZs with elevated levels of BRP. (**A**) Representative images of consecutive evoked release events (green flashes) visualized by expressing myrGCaMP6s in muscle 4. The position of each AZ was determined by expressing GluRIIA-RFP to label the corresponding PSD. Evoked release triggers fusion across different sets of AZs during each stimulus, but a subpopulation of AZs respond more frequently (arrow). (**B**) Histogram of the distribution of AZ *P_r_* for a 0.3 Hz 5 min stimulation paradigm. AZs classified as high *P_r_* (>2 standard deviations above the mean) are shown in red. The percentage of AZs that were low *P_r_* (65.8%), high *P_r_* (9.9%), spontaneous-only (9.7%) and silent (14.6%) is displayed in the inset. (**C**) Average *P_r_* determined for each individual experiment for the AZ population categorized based on low and high activity sites (>2 standard deviations above the mean). Each point represents the average for all AZs (classified as either high or low *P_r_*) from a single animal. (**D**) Individual BRP puncta for three NMJs from three different animals imaged with high resolution structured illumination microscopy (SIM, left panel) or confocal microscopy (middle panel). The right panel displays the heat map for evoked *P_r_* from the same NMJs determined by GCaMP6s imaging prior to fixation. Representative high *P_r_* sites are circled with red in the middle panels. Representative example of a large BRP puncta that would be classified as a single AZ using conventional light microscopy but resolved into multiple clustered AZs using SIM analysis is boxed with the dotted white line. The far right top panel displays the results from the automated detection algorithm that outlines individual AZs. (**E**) AZs were separated into high and low *P_r_* based on their activity and the sum fluorescence intensity of the corresponding BRP puncta is shown. (**F**) AZs with high BRP intensity (two standard deviations above average) were preselected from conventional confocal images and identified on corresponding SIM images. In cases where the BRP signal was resolvable into more than one AZ by SIM microscopy, it was assigned to the AZ cluster group. In cases where the BRP signal mapped to a single BRP puncta by SIM imaging, it was assigned to the single AZ group. *P_r_* is plotted for each group. Student’s t-test was used for statistical analysis (***=p ≤ 0.001). Error bars represent SEM.

Using this approach, we mapped all myrGCaMP6s visualized release events to the position of in vivo GluRIIA-RFP labeled PSDs (Figure 1A). Consistent with previous data, we observed a heterogeneous distribution of AZ *P_r_*, with an average *P_r_* of 0.073 ± 0.002 (n = 1933 AZs from 16 NMJs from 16 animals). However, there was a ~50-fold difference in *P_r_* between the highest and lowest releasing sites. The AZ *P_r_* dataset did not fit a normal distribution (D'Agostino K^2^ test (p<0.0001), Shapiro-Wilk test (p<0.0001), Kolmogorov-Smirnov test (p<0.0001)) and instead was skewed to the right, with a majority of AZs rarely releasing a synaptic vesicle following an action potential (*P_r_* in the range of 0.01 to 0.2) and a small number of AZs consistently showing high release rates (75% percentile of *P_r_* was 0.1, with a maximum *P_r_* of 0.73; Figure 1B). 9.7% of all release sites defined by their apposed GluRIIA receptors displayed only spontaneous fusion events, and another 14.6% of the AZ population was silent for both spontaneous and evoked release during the recording period (Figure 1B). We categorized all AZs with a release rate greater than two standard deviations above average as ‘high *P_r_*’, and the remaining AZs that showed evoked release as ‘low *P_r_*’. Using these criteria, 65.8% of all AZs fell in the low *P_r_* category with an average *P_r_* of 0.049 ± 0.004. In contrast, 9.9% of AZs were classified as high *P_r_* sites, with an average *P_r_* of 0.277 ± 0.015 (Figure 1C). High *P_r_* AZs displayed on average a 5.7-fold higher chance of vesicle fusion following an action potential compared to low *P_r_* AZs.

### High *P_r_* AZs correspond to single release sites with enhanced levels of the AZ protein Bruchpilot

One potential caveat to the interpretation of *P_r_* heterogeneity is the possibility that multiple closely-positioned release sites could be falsely characterized as single high *P_r_* AZs using conventional light microscopy. Presynaptic AZ position can be precisely identified at the NMJ by labeling the core AZ T-bar component Bruchpilot (BRP), the homolog of mammalian ELKS/CAST (Fouquet et al., 2009; Wagh et al., 2006). To determine if the high *P_r_* sites we observed were actually due to release from closely clustered AZs, we used high-resolution structured illumination microscopy (SIM) on fixed tissue stained with anti-BRP (Figure 1D) following dual color (myrGCaMP6s/GluRIIA-RFP) quantal imaging. SIM provides a lateral resolution of ~110 nm (Wegel et al., 2016), providing clear resolution of the BRP ring structure, which has a diameter of ~200 nm (Owald et al., 2012), smaller than the resolution limit of conventional light microscopy. The presence of GluRIIA-RFP allowed us to precisely match individual high *P_r_* sites observed during live imaging with their position in fixed and stained tissue during SIM imaging (Figure 1D). Using an automated detection algorithm in the Volocity 3D image analysis software, we were able to identify all AZs labeled with BRP (Figure 1D, far right panel), and to resolve individual AZ clusters that were not separated using conventional spinning disk microscopy (Figure 1D, white box). SIM analysis of distances between neighboring AZs indicated that 2.45 ± 0.4% (n = 9 NMJs from nine animals) of all AZs were located close enough to each other (within 280 nm) such that they would not be resolvable during live imaging. In contrast, 9.9% (n = 16 NMJs from 16 animals) of AZs were functionally classified as high *P_r_* sites, suggesting that the majority of these sites are not likely to be explained by release from multiple closely clustered AZs.

SIM visualization of BRP at AZs following *P_r_* mapping revealed that a majority of high *P_r_* sites were represented by a single BRP-positive AZ that was not further resolvable after SIM (Figure 1D, red circles). We identified high *P_r_* sites (n = 42 AZs from 5 NMJs from five animals) and then determined what fraction of these sites were truly clusters of multiple neighboring AZs based on their SIM profiles. Only 16 ± 3% of these high *P_r_* sites were resolved into multiple AZs upon SIM analysis, indicating that most high *P_r_* AZs correspond to single release sites. These single BRP clusters at high *P_r_* sites had larger sum fluorescence intensities than most other BRP positive puncta (Figure 1E). The average sum fluorescence of single BRP puncta from high *P_r_* AZs (3.95 × 10^6^ ± 2.67 x 10^5^, n = 24 AZs from 9 NMJs from nine animals) was 1.7-fold greater than the fluorescence of randomly selected low *P_r_* BRP clusters (2.33 × 10^6^ ± 0.98 x 10^5^, n = 60 AZs from 9 NMJs from nine animals, p<0.0001). To further examine large single BRP clusters that could not be resolved using conventional spinning disk microscopy, all BRP clusters larger than 280 nm were automatically detected and assigned their *P_r_* measured during live imaging. We then determined whether these sites were represented by single or multiple AZs using SIM microscopy. Clusters > 280 nm in diameter that could be resolved to multiple BRP positive AZs after SIM imaging had a lower *P_r_* (0.10 ± 0.02, n = 35 AZs from 5 NMJs from five animals) than those comprised of a single large BRP positive AZ (0.19 ± 0.02, n = 42 AZs from 5 NMJs from five animals; Figure 1F). As such, high resolution SIM analysis confirms that most high *P_r_* sites correspond to single AZs with more intense BRP labeling, consistent with previous data supporting the positive role of BRP in regulating *P_r_* (Paul et al., 2015; Peled et al., 2014; Reddy-Alla et al., 2017).

### Release heterogeneity is retained in *Synaptotagmin one* mutants

**Figure 2.**
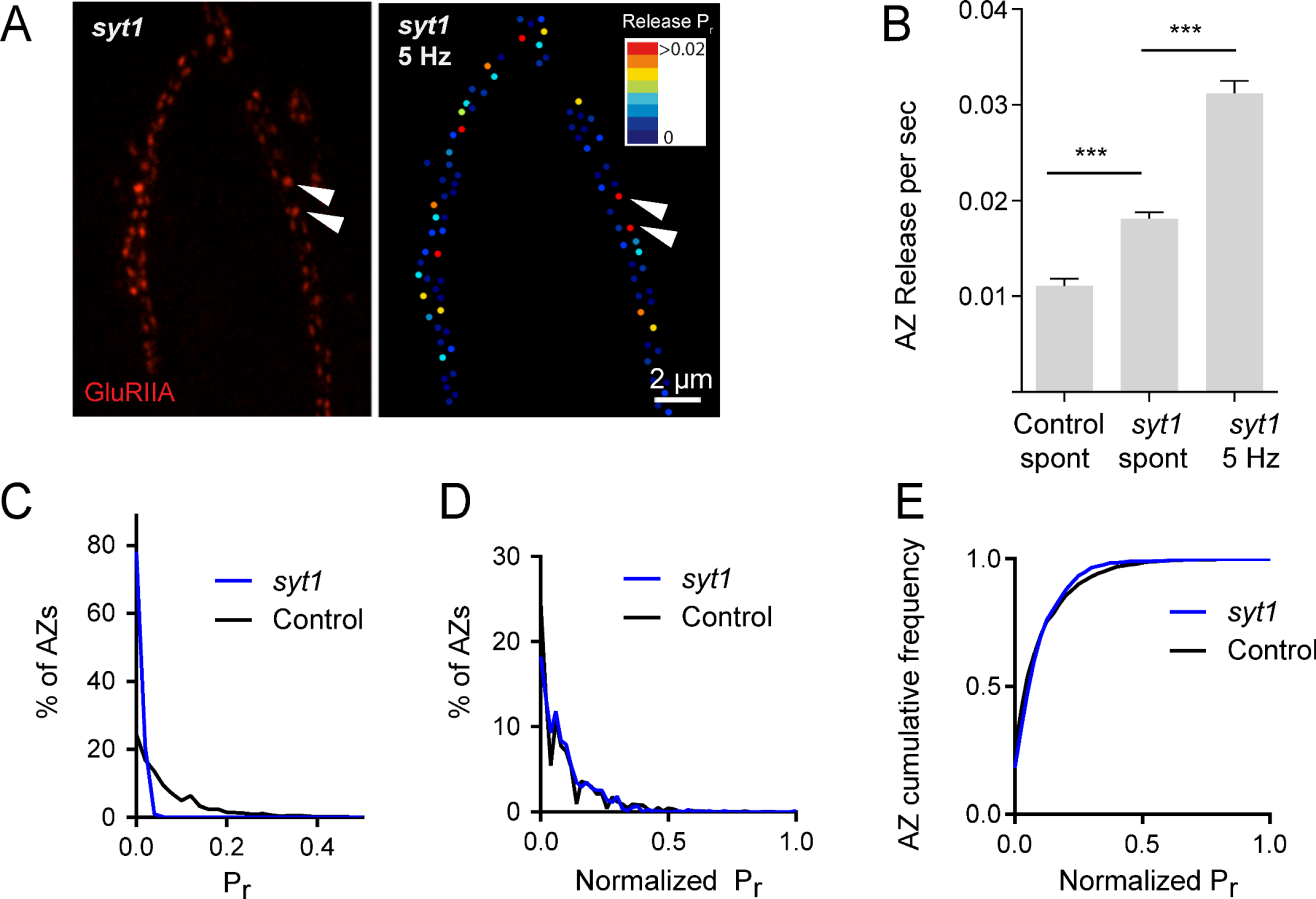
*P_r_* variability remains in *syt1* null mutants. (**A**) The left panel displays the distribution of GluRIIA in *syt1* nulls (left panel) at the muscle 4 NMJ. The corresponding *P_r_* heatmap is shown on the right. The arrows denote several high *P_r_* sites opposed by bright GluRIIA positive PSDs. (**B**) AZ release events per second for spontaneous release and evoked by 5 Hz stimulation are shown for *syt1* nulls mutants, and for spontaneous release in controls. (**C**) Frequency distribution of *P_r_* is shown for *syt1* nulls and controls. (**D**) Plot of normalized *P_r_* frequency distribution (from 0 to 1 (max)) for *syt1* nulls and controls. (**E**) Cumulative frequency distribution for normalized release rates for *syt1* nulls and controls is shown. One-way ANOVA followed by Dunnett’s multiple comparisons test used for statistical analysis (***=p ≤ 0.001). Error bars represent SEM.

Heterogeneous release rates between AZs could solely reflect stable differences in protein content of the AZs themselves. However, the accumulation of different synaptic vesicle populations with variable levels of Ca^2+^ sensitivity or fusogenicity might also contribute to release heterogeneity. The synchronous Ca^2+^ sensor Synaptotagmin 1 (Syt1) resides on synaptic vesicles and plays a major role in *P_r_* determination at *Drosophila* NMJs (DiAntonio and Schwarz, 1994; Guan et al., 2017; Lee et al., 2013; Littleton et al., 1994, 1993; Yoshihara et al., 2003; Yoshihara and Littleton, 2002). We hypothesized that if differential synaptic vesicle Ca^2+^ sensitivity is a major determinant of release heterogeneity in addition to its established role in determining overall *P_r_*, then elimination of Syt1 would disrupt *P_r_* heterogeneity. Consistent with electrophysiological findings, quantal imaging in *syt1* null mutants expressing GluRIIA-RFP and myrGCaMP6s revealed a dramatic reduction in evoked release, a shift from synchronous to highly asynchronous fusion, and an increase in spontaneous release rates (Video 2). To estimate AZ release heterogeneity in *syt1* nulls, preparations were stimulated at 5 Hz and release events were normalized to the number of stimuli (Figure 2A). The average release rate per AZ per second in *syt1* nulls during 5 Hz stimulation was 0.03 ± 0.001 (n = 719 AZs from 7 NMJs from six animals; Figure 2B). In contrast, spontaneous release rate per AZ in the absence of stimulation was 0.018 ± 0.001 per second in *syt1* nulls (n = 719 AZs from 7 NMJs from six animals) compared to 0.011 ± 0.001 in controls (n = 559 AZs from 6 NMJs from four animals, p<0.0001; Figure 2B).

**Video 2.** Representative movie showing spontaneous GCaMP6s events in *syt1* mutants expressing GluRIIA-RFP (red), followed by GCaMP6s events observed during 5 Hz stimulation.

Although release rate is dramatically reduced in *syt1* nulls, AZs still maintained overall heterogeneity in *P_r_* distribution. Comparing the distribution of AZ release rates for *syt1* nulls and controls, release was proportionally decreased across all AZs in *syt1* (Figure 2C); frequency distribution analysis of AZs with normalized release rates (from 0 to maximum release) confirmed that there was no significant change in the heterogeneity of release between *syt1* mutants and controls (Figure 2D,E). Given that AZ *P_r_* remains highly heterogeneous in the absence of Syt1, these data indicate that heterogeneity in synaptic vesicle Ca^2+^ sensitivity between AZs is unlikely to play a major role in *P_r_* distribution.

### Individual AZ *P_r_* remains stable through extensive vesicle cycling

**Figure 3.**
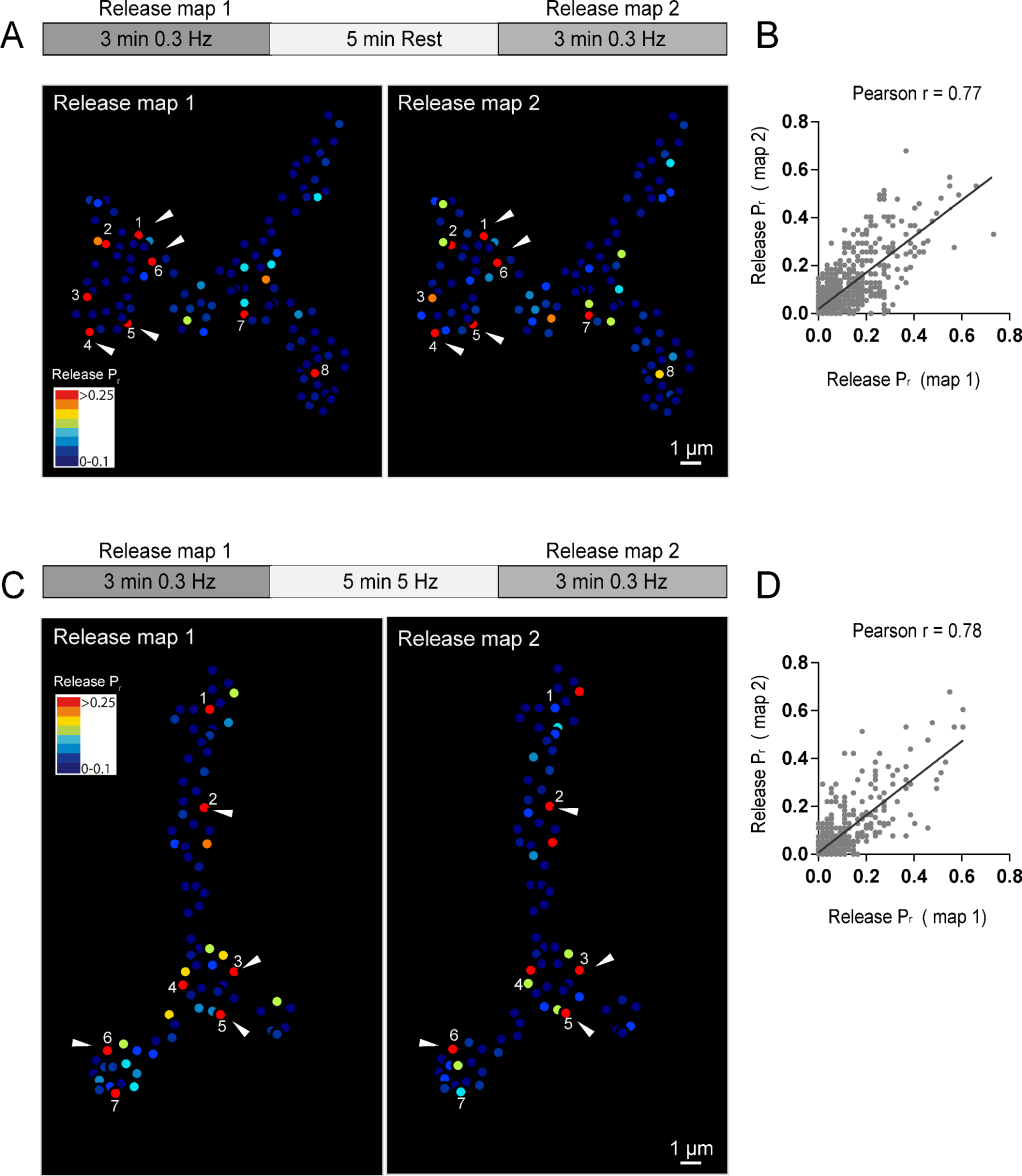
Stability of release maps at the NMJ. (**A**) *P_r_* heatmaps for the same muscle 4 NMJ were generated for two individual imaging sessions, separated by a 5 min resting period. High *P_r_* AZs were numbered and re-identified in each heatmap. Representative high *P_r_* AZs that sustain release rates during the second imaging session are noted with arrows. (**B**) Correlation of AZ *P_r_* between two imaging sessions separated by a 5 min resting period. (**C**) *P_r_* heatmaps for the same NMJ separated by a 5 min 5 Hz stimulation. Representative high *P_r_* AZs that did not change activity levels are noted with arrows. (**D**) Correlation of AZ *P_r_* between two imaging sessions separated by a 5 min 5 Hz stimulation period.

Release heterogeneity in *syt1* null animals suggests that synaptic vesicle Ca^2+^ sensitivity is not a major determinant of *P_r_* heterogeneity at this synapse; however, it is possible that other synaptic vesicle components influence *P_r_* on an AZ-specific level. If unique local synaptic vesicle pools contribute to the distribution of *P_r_* between AZs, we predicted that *P_r_* would be highly dynamic at individual AZs over time. In contrast, stability of *P_r_* would argue that release heterogeneity is more likely associated with stably resident proteins at individual AZs. To assess *P_r_* stability over time, we conducted a 3 min imaging session using 0.3 Hz stimulation to generate an initial *P_r_* map, and then allowed the preparation to rest for 5 min without stimulation or imaging before re-mapping *P_r_* in a final 3 min imaging session. We were constrained in our ability to examine *P_r_* continuously over longer time intervals due to bleaching of GCaMP6s from the high frequency capture rate. *P_r_* at individual AZs was very stable between the two sessions (Figure 3A). This was especially evident for high *P_r_* sites, which sustained high levels of activity during both imaging sessions. Plotting release rate for all AZs revealed a strong correlation for *P_r_* across the two imaging sessions (Pearson r = 0.77, R^2^ = 0.59, p<0.0001, n = 988 AZs from 8 NMJs from seven animals; Figure 3B).

We next used a strong stimulation paradigm to drive vesicle cycling to promote intermixing of synaptic vesicles. NMJ preparations were imaged during two low frequency 0.3 Hz stimulation periods separated by a 5 min 5 Hz stimulation session (Figure 3C). Release maps were not dramatically altered by 5 Hz stimulation, with the overall correlation of *P_r_* between the two imaging sessions similar with and without stimulation (Pearson r = 0.78, R^2^ = 0.61, p<0.0001, n = 613 AZs from 6 NMJs from six animals; Figure 3D). Thus, inducing vesicle recycling with 5 Hz stimulation does not dramatically change *P_r_* across the AZ population, arguing that stably resident AZ components, rather than AZ-specific synaptic vesicle populations, are likely to represent the major driver of *P_r_* heterogeneity at this synapse.

### *P_r_* is correlated with Ca^2+^ channel abundance at AZs

Given that vesicle fusion is highly sensitive to Ca^2+^ and most effective in close proximity to VGCCs (Augustine et al., 1985; Böhme et al., 2016; Chen et al., 2015; Heidelberger et al., 1994; Katz and Miledi, 1967; [Bibr bib49][Bibr bib124]; Keller et al., 2015; Meinrenken et al., 2003, 2002; Nakamura et al., 2015; Sheng et al., 2012; Stanley, 2016; Wang et al., 2008), Ca^2+^ channel abundance at individual AZs is predicted to be a key variable for *P_r_* heterogeneity at *Drosophila* NMJs as well. Cacophony (*cac*) encodes the *Drosophila* voltage-activated Ca^2+^ channel α1 subunit required for neurotransmitter release (Fouquet et al., 2009; Kawasaki et al., 2004; 2000; Littleton and Ganetzky, 2000; Liu et al., 2011; Rieckhof et al., 2003; Smith et al., 1996). Transgenic animals expressing fluorescently tagged Cac channels have been previously generated, demonstrating that Cac localizes specifically to AZs and its abundance appears heterogenous across release sites (Kawasaki et al., 2004; Matkovic et al., 2013; Yu et al., 2011).

To examine the heterogeneity of Cac abundance across AZs, we used SIM to measure the distribution of Cac-GFP and BRP at muscle 4 NMJs. Using this approach, we observed a heterogeneous distribution of mean Cac-GFP fluorescence at AZs, similar to the variable levels of BRP intensity described earlier (Figure 1D, Figure 4—figure supplement 1A,B). 5.72% of AZs displayed Cac-GFP fluorescence greater than two standard deviations above average (n = 2011 AZs from 11 NMJs from three animals). The mean Cac-GFP fluorescence for these bright AZs was 2.1-fold greater than that observed for the remaining sites (p<0.0001; Figure 4—figure supplement 1C). Mean AZ intensities of Cac-GFP and BRP were positively correlated (Pearson r = 0.46, R^2^ = 0.21, p<0.0001, n = 730 AZs from 6 NMJs from three animals; Figure 4—figure supplement 1D,E).

**Figure 4.**
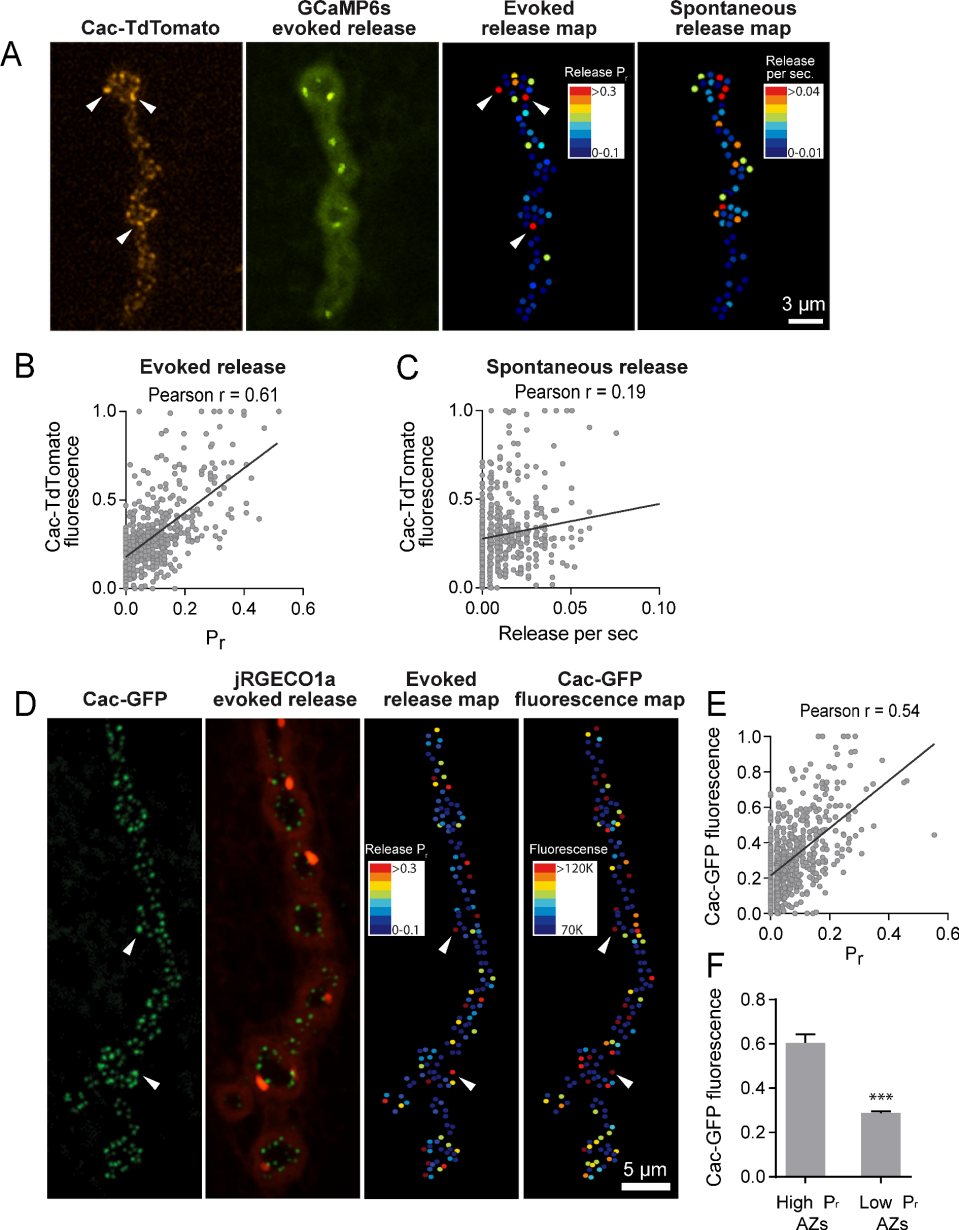
*P_r_* correlates with Cac channel abundance at AZs. (**A**) Representative images showing heterogeneous distribution of Cac-TdTomato at the NMJ of muscle 4 (left panel). Evoked release was visualized at the same NMJ using myrGCaMP6s (second panel) and AZ release maps were generated for evoked (third panel) and spontaneous fusion (right panel). Several high *P_r_* AZs with bright Cac density are noted (arrows). (**B**) Correlation between AZ *P_r_* and Cac-TdTomato fluorescence intensity. (**C**). Correlation between AZ spontaneous release rate per second and Cac-TdTomato fluorescence intensity. (**D**) Representative images showing heterogeneous distribution of Cac-GFP at the NMJ (left panel). Evoked release visualized at the same NMJ by myr-jRGECO1a is shown in the second panel. The *P_r_* heatmap for evoked release is shown in the third panel. A heatmap distribution of Cac-GFP fluorescence intensities, based on same two standard deviation criteria as color-coding of *P_r_*, is shown in the right panel. The arrows denote several higher *P_r_* sites that correlated with bright Cac-GFP puncta. (**E**) Correlation between AZ *P_r_* and Cac-GFP fluorescence intensity for evoked release. (**F**) Cac-GFP fluorescence for AZs functionally classified as either low or high *P_r_* (>2 standard deviations above mean) by quantal imaging with myr-jRGECO1a. Student’s t-test was used for statistical analysis (***=p ≤ 0.001). Error bars represent SEM.

To determine whether Cac distribution correlates with *P_r_* heterogeneity, we used dual color imaging experiments where vesicle fusion events were detected by myrGCaMP6s driven in muscles using mef2-GAL4 and Ca^2+^ channel distribution was visualized using red-labeled Cac-TdTomato expressed pan-neuronally using elav-GAL4 (Figure 4A). We observed a strong positive correlation (average Pearson r = 0.61, R^2^ = 0.38, p<0.0001, n = 483 AZs from 7 NMJs from seven animals) between AZ Cac fluorescence intensity and evoked AZ *P_r_* (Figure 4B). In contrast, single AZ release rates for spontaneous events showed only a mild correlation with Cac intensity (average Pearson r = 0.19, R^2^ = 0.036, p<0.0001, n = 483 AZs from 7 NMJs from seven animals; Figure 4C). These results are consistent with previous observations that release rates for evoked and spontaneous fusion are not correlated at *Drosophila* AZs (Melom et al., 2013; Peled et al., 2014), and that spontaneous fusion is largely independent of extracellular Ca^2+^ at this synapse (Jorquera et al., 2012; Lee et al., 2013).

**Figure 4—figure supplement 1.**
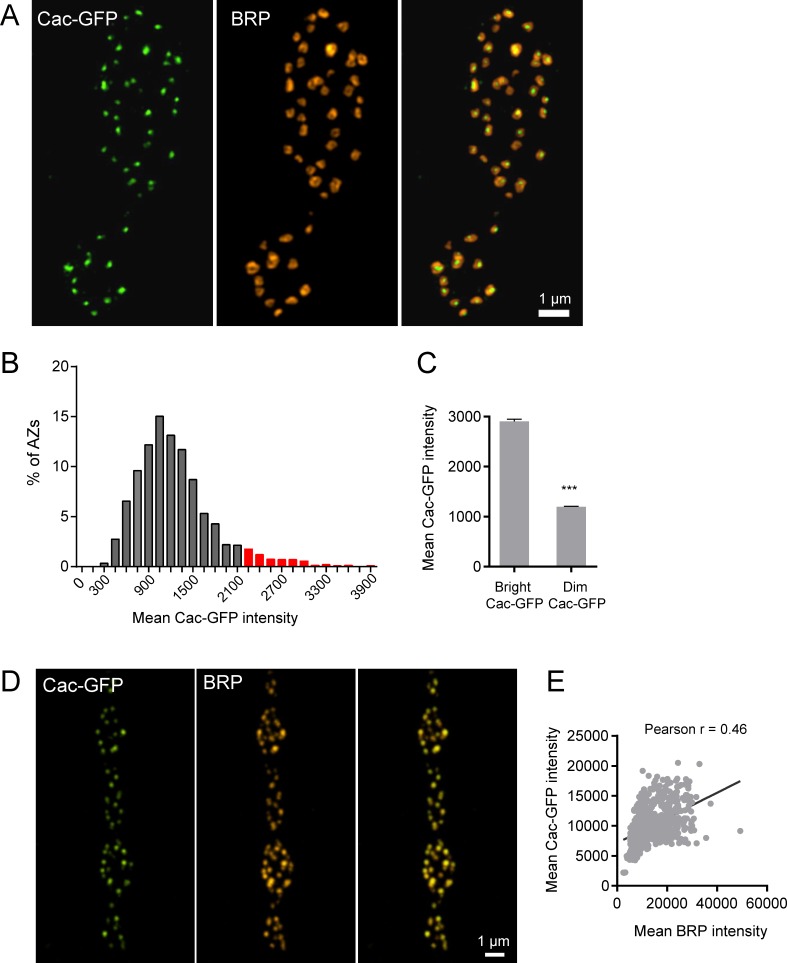
Cac-GFP distribution at AZs analyzed by SIM microscopy. (**A**) Representative Cac-GFP and BRP puncta at AZs for two synaptic boutons imaged using SIM microscopy. (**B**) Histogram of the distribution of mean Cac-GFP fluorescence intensity across the AZ population. Red corresponds to the Cac-GFP containing AZ population with fluorescence intensity two standard deviations above the mean. (**C**) Mean fluorescence intensity of Cac-GFP for bright (fluorescence greater than two standard deviations above the average) versus dim AZs. Student’s t-test was used for statistical analysis (***=p ≤ 0.001). Error bars represent SEM. (**D**) Representative images of Cac-GFP and BRP (stained using anti-BRP antibody) in fixed tissue, imaged using conventional light microscopy. (**E**) Correlation between mean Cac-GFP fluorescence intensity and mean BRP fluorescence intensity imaged using conventional light microscopy.

To increase confidence that the observed Cac-TdTomato intensity accurately reflects Cac distribution, we also measured the correlation between *P_r_* and Cac channels transgenically tagged with GFP. We generated transgenic lines expressing myristoylated red Ca^2+^ indicators previously characterized in the field, including RCaMP1h, R-GECO1 and jRGECO1a. Although RCaMP1h and R-GECO1 were too dim to visualize localized Ca^2+^ transients at PSDs, transgenic lines expressing the myristoylated Ca^2+^ sensor jRGECO1a (Dana et al., 2016) in muscle four allowed detection of Ca^2+^ influx following vesicle fusion at single AZs (Video 3). In contrast to the more robust GCaMP6s, jRGECO1a has a shorter fluorescent lifetime and the signal amplitude decays more rapidly. We observed that quantal events imaged with myr-jRGECO1a were dimmer and fully bleached within 7–10 min of imaging. Therefore, preparations were stimulated at 1 Hz for shorter two-minute imaging sessions to generate *P_r_* maps in larvae expressing myr-jRGECO1a (Figure 4D). We observed a strong correlation between AZ *P_r_* detected by myr-jRGECO1a and Cac-GFP intensity (average Pearson r = 0.54, R^2^ = 0.29, p<0.0001, n = 651 AZs from 7 NMJs from seven animals, correlation from a representative experiment shown in Figure 4E). Again, we found a weaker correlation between spontaneous fusion and Cac-GFP intensity (Pearson r = 0.17, R^2^ = 0.03, p<0.0001, n = 651 AZs from 6 NMJs from six animals). Hence, *P_r_* for action-potential evoked fusion is strongly correlated with the local abundance of Cac channels at individual *Drosophila* AZs.

**Video 3.** Representative movie showing evoked and spontaneous jRGECO events (red) in larvae expressing Cac-GFP (green).

We next compared Cac-GFP fluorescence at AZs that were functionally classified as either low or high *P_r_* sites by quantal imaging using myr-jRGECO1a (Figure 4F). The average fluorescence of single Cac-GFP puncta from high *P_r_* AZs (normalized intensity = 0.6 ± 0.04, n = 38 AZs from 7 NMJs from seven animals) was 2.09-fold greater than the average fluorescence of low *P_r_* AZs (normalized intensity = 0.29 ± 0.01, n = 638 AZs from 7 NMJs from seven animals, p<0.0001). We also examined the *P_r_* of AZs classified by Cac-GFP fluorescence. The average *P_r_* for AZs displaying high Cac-GFP fluorescence (>2 standard deviations above average) was 0.2 ± 0.016 (n = 7 NMJs from seven animals) compared to 0.06 ± 0.003 (n = 7 NMJs from seven animals, p<0.0001) for the remaining AZs with lower levels of Cac-GFP. Although the absolute number of Cac channels at single *Drosophila* AZs is unknown, these data suggest that a ≥ 2 fold difference in channel number exists between low and high *P_r_* AZs. Given the steep third to fourth order non-linear dependence of synaptic vesicle fusion on Ca^2+^ (Dodge and Rahamimoff, 1967; Heidelberger et al., 1994; Jan and Jan, 1976), a small change in channel number is likely to have a large effect on *P_r_*.

### *P_r_* correlates with the level of presynaptic Ca^2+^ influx at individual AZs

**Figure 5.**
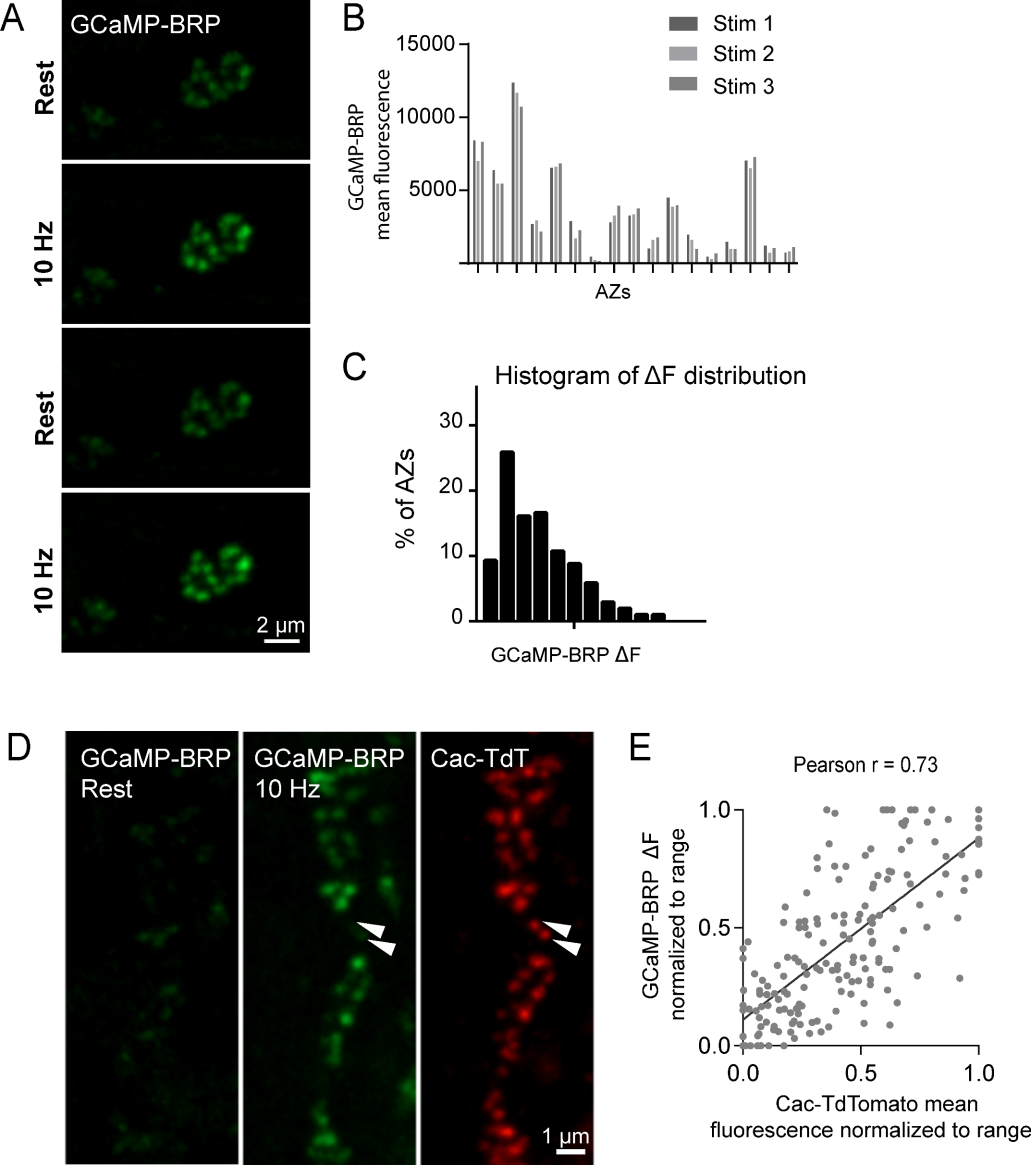
GCaMP-BRP detects relative Ca^2+^ influx at single AZs and is correlated with Cac channel abundance. (**A**) Representative images of the same muscle 4 NMJ bouton showing CCaMP6m-BRP fluorescence at rest and following 10 Hz stimulation for two consecutive rounds. (**B**) The AZ fluorescence intensity was plotted for three independent rounds of stimulation for BRP-GCaMP6m. Fluorescence changes per AZ remain stable for the same AZ during multiple rounds of stimulation. (**C**) Histogram of the distribution of relative fluorescence intensities (ΔF) across AZs for BRP-GCaMP6m. (**D**) Representative images showing GCaMP6m-BRP fluorescence before (left panel) and during stimulation (middle panel). The corresponding distribution of Cac channels labeled by Cac-TdTomato is shown for the same NMJ (right panel). Examples of rare Cac-positive AZs that showed no corresponding Ca^2+^ influx are indicated (arrows). (**E**) Correlation between GCaMP6m-BRP ΔF during stimulation and Cac-TdTomato fluorescence intensity at individual AZs.

VGCCs are extensively modulated by second messenger pathways that can alter channel conductivity (Catterall and Few, 2008; Dolphin et al., 1991; Evans and Zamponi, 2006; Reid et al., 2003; Tedford and Zamponi, 2006; Zamponi and Snutch, 1998). Although Cac channel abundance correlates with AZ *P_r_*, an important readout of channel activity is the local Ca^2+^ influx occurring at each AZ. Assaying presynaptic Ca^2+^ influx directly would also be useful to bypass any unknown effects on *P_r_* generated by expressing fluorescently tagged Ca^2+^ channels. As such, we generated transgenic animals expressing GCaMP6m fused to the N-terminus of BRP, which localizes directly to the base of the AZ where Ca^2+^ channels cluster (Fouquet et al., 2009; Kittel et al., 2006). These GCaMP6m fusions were made to a BRP fragment (BRP^short^) corresponding to amino acids 473–1226 of the full 1740 amino acid protein (Schmid et al., 2008). At rest, N-terminal GCaMP-BRP^short^ was dim, consistent with low levels of resting Ca^2+^ (Figure 5A). Stimulation at 10 Hz resulted in a robust increase in discrete punctated presynaptic fluorescence that remained confined to single AZs during stimulation (Figure 5A). During multiple rounds of 5 s 10 Hz stimulation, GCaMP-BRP^short^ fluorescence increase (ΔF) varied between AZs, but was stable at the same AZ for each independent stimulation (n = 205 AZs from 6 NMJs from three animals; Figure 5B,C).

**Figure 5—figure supplement 1.**
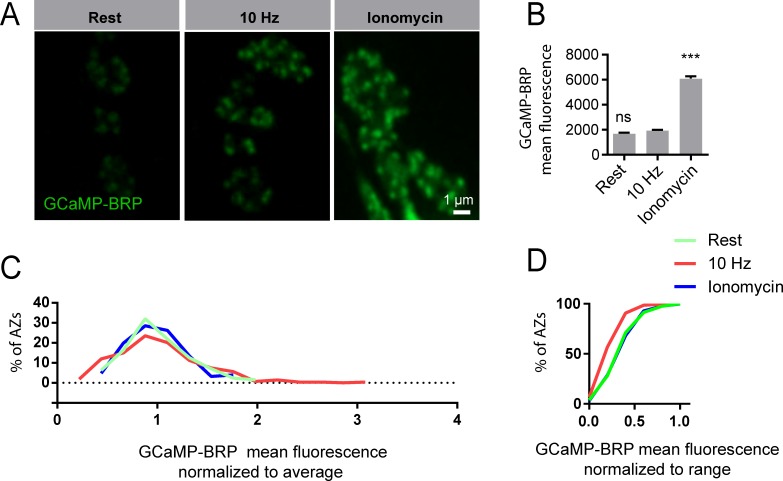
Properties of GCaMP-BRP during stimulation and ionomyocin application. (**A**) Representative images of GCaMP6s-BRP^short^ fluorescence in 1.3 mM Ca^2+^ at rest (no stimulation), during 10 Hz stimulation, and upon 200 nM ionomycin application. (**B**) GCaMP-BRP^short^ mean fluorescence at rest, during 10 Hz stimulation, and after ionomycin application. One-way ANOVA followed by Dunnett’s multiple comparisons test was used for statistical analysis (***=p ≤ 0.001). Error bars represent SEM. (**C, D**) Distribution of mean AZ fluorescence intensity normalized to average (**C**) and cumulative frequency distribution of mean AZ fluorescence normalized to range (**D**) of GCaMP6s-BRP^short^ at rest (green), during 10 Hz stimulation (red) and following ionomycin application (blue).

Given that GCaMP-BRP^short^ abundance at an AZ likely reflects the absolute amount of BRP at that AZ, we assayed if heterogeneity in GCaMP-BRP^short^ fluorescence during 10 Hz stimulation could be solely explained by differences in sensor distribution across AZs. We applied 200 nM of the Ca^2+^ ionophore ionomycin to elevate Ca^2+^ concentrations uniformly throughout the terminal independent of Cac abundance. In the presence of ionomycin, differences in fluorescent signals between AZs should be entirely due to heterogeneity in sensor abundance. We observed a rightward shift in the GCaMP-BRP^short^ intensity distribution among AZs upon ionomycin application compared to 10 Hz stimulation (average AZ fluorescence during 10 Hz stimulation was 1924 ± 63, and following ionomycin addition was 6105 ± 175; Figure 5—figure supplement 1A,B), indicating that during 10 Hz stimulation, detection of Ca^2+^ by GCaMP-BRP^short^ is not limited by sensor abundance. Furthermore, we observed a significant difference in the shape of the distribution during 10 Hz stimulation compared to both before stimulation and after ionomycin. The distribution of fluorescence intensities is narrower both at rest and upon ionomycin application; these two distributions should primarily reflect sensor distribution. In contrast, the distribution of GCaMP-BRP^short^ fluorescence during 10 Hz stimulation is wider, indicating that the sensor is reporting local changes in Ca^2+^ influx and not just sensor distribution (Figure 5, Figure 5—figure supplement 1C,D). Thus, although GCaMP-BRP^short^ abundance is likely to contribute to the levels of Ca^2+^ influx detected, these results are consistent with heterogeneity in Ca^2+^ influx across individual AZs.

**Figure 6.**
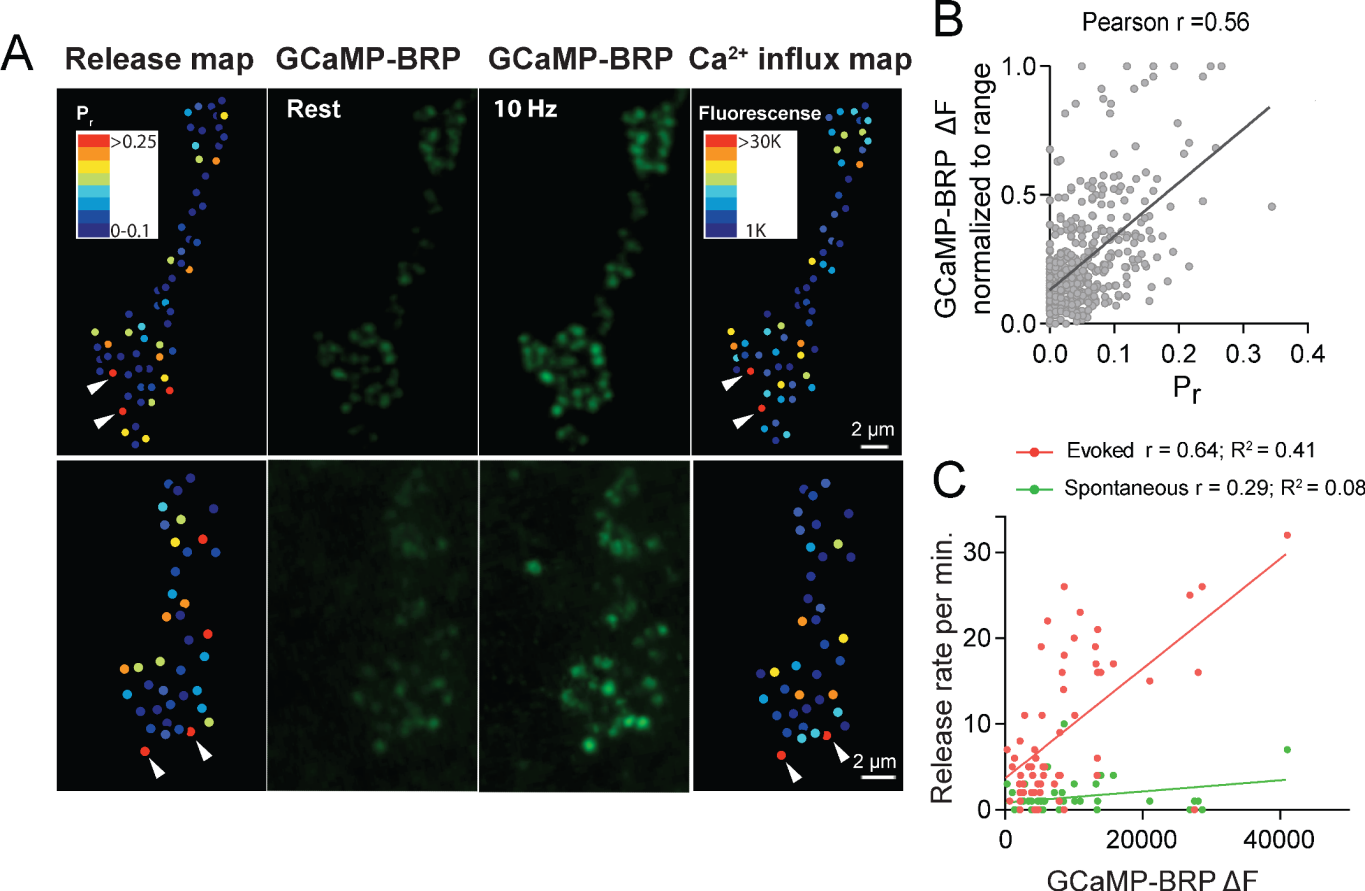
*P_r_* correlates with the relative levels of Ca^2+^ influx at AZs. (**A**) Two representative muscle 4 NMJs with AZ *P_r_* heatmaps obtained following myr-jRGECO1a mapping during stimulation (left panel). GCaMP6m-BRP^short^ fluorescence levels of the same NMJ at rest (second panel) and during stimulation (third panel) are shown. Heatmaps of GCaMP6m-BRP^short^ ΔF during stimulation are displayed in the right panel. Several representative high *P_r_* AZs that experienced the strongest Ca^2+^ influx detected by GCaMP6m-BRP^short^ are noted (arrows). (**B**) Correlation between GCaMP6m-BRP^short^ ΔF (during 10 Hz stimulation) and AZ *P_r_* (during 1 Hz stimulation) is shown across all experiments. (**C**) Representative correlation between GCaMP6m-BRP ΔF and AZ release rate per minute for evoked (red) and spontaneous (green) fusion for a representative single NMJ.

We next assayed if Ca^2+^ influx detected by GCaMP-BRP^short^ is correlated with Cac channel abundance. Animals expressing both Cac-TdTomato and GCaMP-BRP^short^ transgenes in the presynaptic compartment displayed a strong correlation between Ca^2+^ dependent excitation of GCaMP-BRP^short^ (ΔF) and Cac-TdTomato intensity at individual AZs during stimulation (Pearson r = 0.73, R^2^ = 0.53, p<0.0001, n = 176 AZs from 7 NMJs from six animals; Figure 5D,E). We observed a weaker correlation between Cac intensity and GCaMP-BRP^short^ ΔF at rest (Pearson r = 0.18, R^2^ = 0.03, p<0.001, n = 338 AZs from 8 NMJs from six animals). A few instances were noted where specific AZs experienced a disproportionally low GCaMP-BRP^short^ ΔF signal relative to their Cac-TdTomato intensity (Figure 5D, arrows), suggesting Ca^2+^ influx may be fine-tuned at certain release sites. We next analyzed the correlation between GCaMP-BRP^short^ ΔF induced by 10 Hz stimulation and release rate visualized by postsynaptic myr-jRGECO1a during 1 Hz stimulation (Figure 6A,B). AZs that experienced stronger Ca^2+^ influx displayed higher *P_r_* during stimulation. Overall, there was a strong correlation between Ca^2+^ influx and AZ *P_r_* (Pearson r = 0.56, R^2^ = 0.31, p<0.0001, n = 492 AZs from 6 NMJs from six animals; Figure 6B). In contrast, the frequency of spontaneous vesicle fusion per AZ was only mildly correlated with GCaMP-BRP^short^ ΔF (Pearson r = 0.23, R^2^ = 0.07, n = 492 AZs from 6 NMJs from six animals; correlations from a representative experiment shown in Figure 6C). It is worth noting that although a strong correlation between Ca^2+^ influx and evoked *P_r_* was observed at most AZs, a minority population of release sites that displayed robust Ca^2+^ influx had very low *P_r_* (Figure 6B).

**Figure 6—figure supplement 1.**
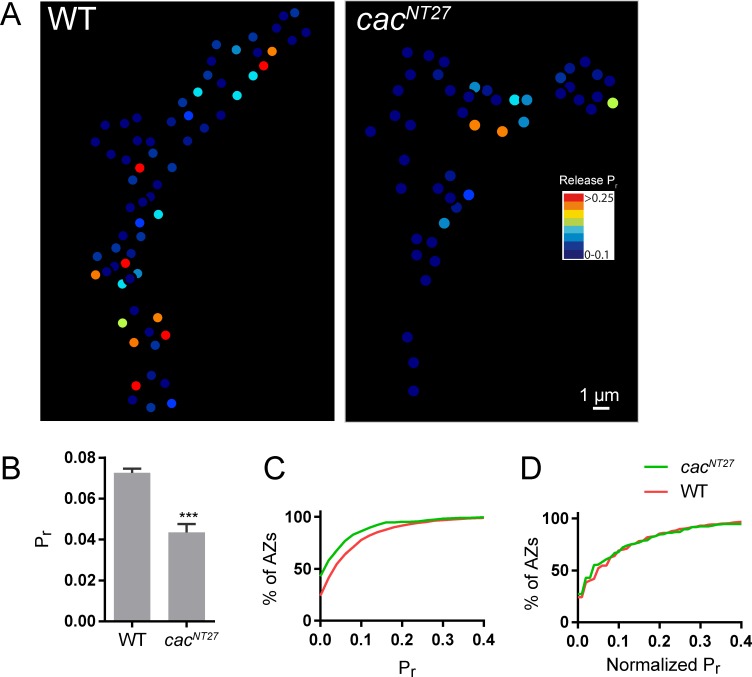
Release probability mapping in the *cac^NT27^* mutant. (**A**) Evoked *P_r_* heatmaps for muscle 4 NMJs were generated in control (left) and *cac^NT27^* (right) third instar larvae. (**B**) Average *P_r_* in control and *cac^NT27^* NMJs. Student’s t-test was used for statistical analysis (***=p ≤ 0.001). Error bars represent SEM. (**C**) Cumulative frequency distribution of *P_r_* for control and *cac^NT27^* AZs. (**D**) Cumulative frequency distribution of *P_r_*, normalized from 0 to max *P_r_* for control and *cac^NT27^* NMJs.

To functionally test if the level of Ca^2+^ influx rather than the structural presence of the Ca^2+^ channel is responsible for determining *P_r_*, we generated *P_r_* maps in the *cac^NT27^* mutant using dual color quantal imaging with GluRIIA-RFP and myr-GCamP6s (Figure 6—figure supplement 1A). Cac^NT27^ channels have reduced Ca^2+^ conductance due to a point mutation in the S4 voltage sensor (Rieckhof et al., 2003). We observed that *cac^NT27^* results in a global decrease in *P_r_* across AZs; evoked *P_r_* in controls ranged from 0 to 0.73 with an average of 0.073 ± 0.0021 (n = 1933 AZs from 16 NMJs from 16 animals), while *cac^NT27^ P_r_* ranged from 0 to 0.47 with a significantly lower average *P_r_* of 0.049 ± 0.0045 (n = 275 AZs from 5 NMJs from five animals; Figure 6—figure supplement 1B). While the entire *P_r_* distribution was shifted lower compared to controls (Figure 6—figure supplement 1C), the normalized distribution of *P_r_* was nearly identical to controls (Figure 6—figure supplement 1D). These findings indicate that the levels of Ca^2+^ influx through Cac channels, rather than the physical presence of the channels, is a primary determinant of *P_r_*.

### Segregation of postsynaptic glutamate receptor subunits at high *P_r_* AZs

**Figure 7.**
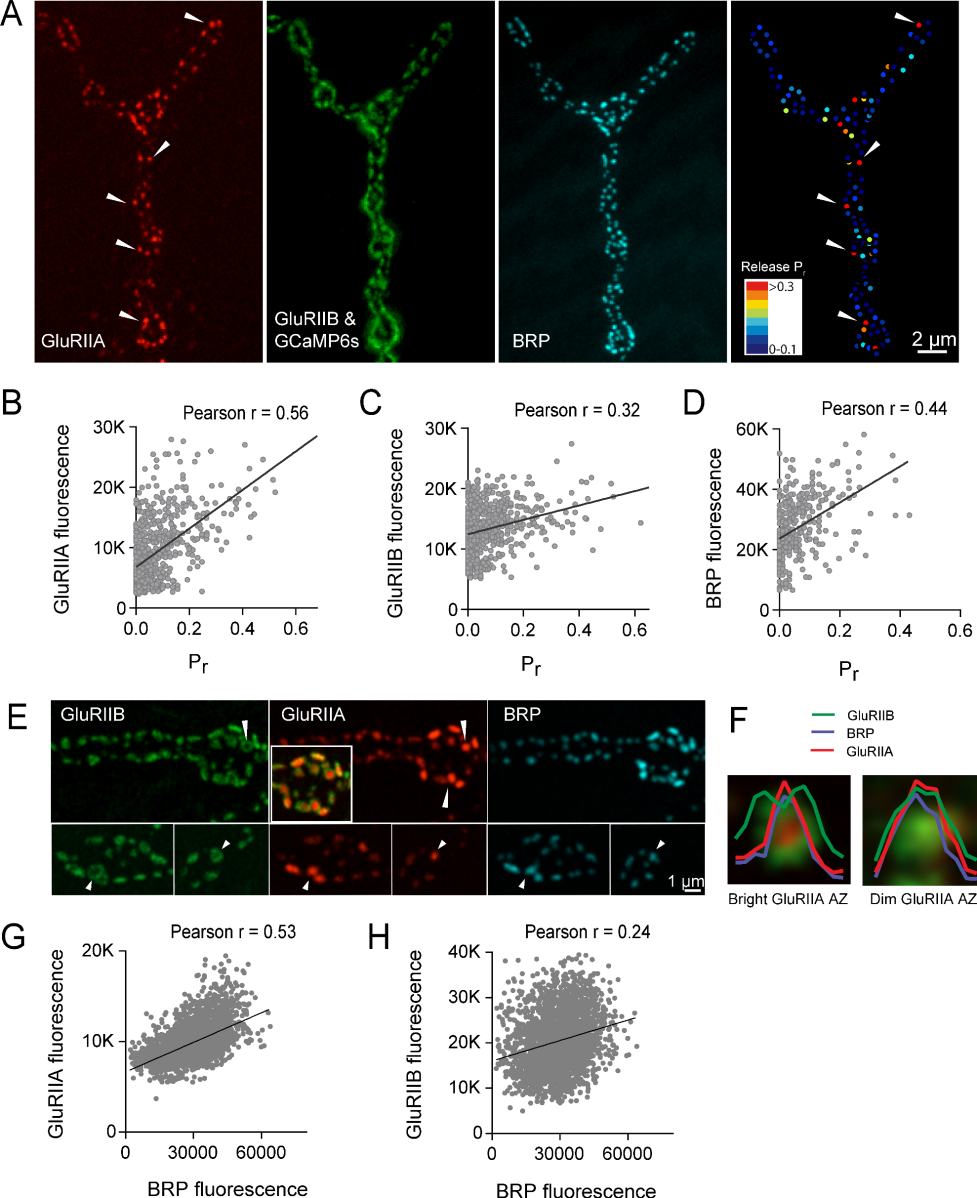
High *P_r_* AZs have elevated PSD GluRIIA levels and display a distinct pattern of glutamate receptor clustering. (**A**) Representative image showing the heterogeneous distribution of GluRIIA-RFP (left panel) at a third instar muscle 4 NMJ. More uniform GluRIIB-GFP PSD puncta can also be observed over the much dimmer myrGCaMP6s (second panel). BRP distribution (third panel) and *P_r_* heatmaps (right panel) for the same NMJ are shown. Several bright GluRIIA fields (intensity two standard deviations above average) are marked with white arrows. The correlation between AZ *P_r_* and GluRIIA-RFP (**B**), GluRIIB (**C**) and BRP (**D**) fluorescence intensity is plotted. (**E**) Representative images showing distribution of GluRIIA, GluRIIB and BRP, without co-expression of myrGCaMP6s. Synapses containing bright GluRIIA puncta have GluRIIB predominantly localized to the periphery of the PSD (arrows), surrounding a GluRIIA core. These AZs have higher BRP intensities as well. (**F**) Average fluorescence line profiles showing GluRIIA, GluRIIB and BRP normalized to fluorescence range across average PSDs, separated into two groups according to their GluRIIA brightness, with ‘bright’ PSDs based on their GluRIIA intensity (two standard deviations above average). The peripheral distribution of GluRIIB around central GluRIIA cores was most obvious for bright GluRIIA-positive PSDs that were shown to be more active during stimulation. Correlation between GluRIIA-RFP (**G**) or GluRIIB-GFP (**H**) with BRP intensity at individual AZs.

We next examined if postsynaptic glutamate receptor composition varied at low *P_r_* versus high *P_r_* AZs. *Drosophila* glutamate receptors at the NMJ assemble as heteromeric tetramers containing three essential subunits (GluRIII, IID and IIE) and a variable fourth subunit of GluRIIA or GluRIIB, with the GluRIIA subtypes having a higher conductance than GluRIIB (Featherstone et al., 2005; Marrus et al., 2004; Petersen et al., 1997; Qin et al., 2005; Schuster et al., 1991). To determine if the GluR subtypes differentially accumulate at AZs in a manner that correlates with presynaptic *P_r_*, we visualized GluRIIA-RFP and GluRIIB-GFP expressed under the control of their endogenous promoters (Rasse et al., 2005). To image myrGCaMP6s activity without obscuring GluRIIB-GFP, myrGCaMP6s was expressed at low levels using the LexA/LexOP system in muscle four with Mef2-LexA. LexA driven myrGCaMP6s signal is dimmer than UAS-myrGCaMP6s at rest and does not obscure the brighter GluRIIB-GFP PSD puncta (Figure 7A). However, upon Ca^2+^ binding to myrGCaMP6s, the fluorescence dramatically increases compared to the level of endogenous GluRIIB-GFP signal, allowing simultaneous imaging of baseline GluRIIB-GFP levels and synaptic activity detected by myrGCaMP6s (Video 4).

**Figure 7—figure supplement 1.**
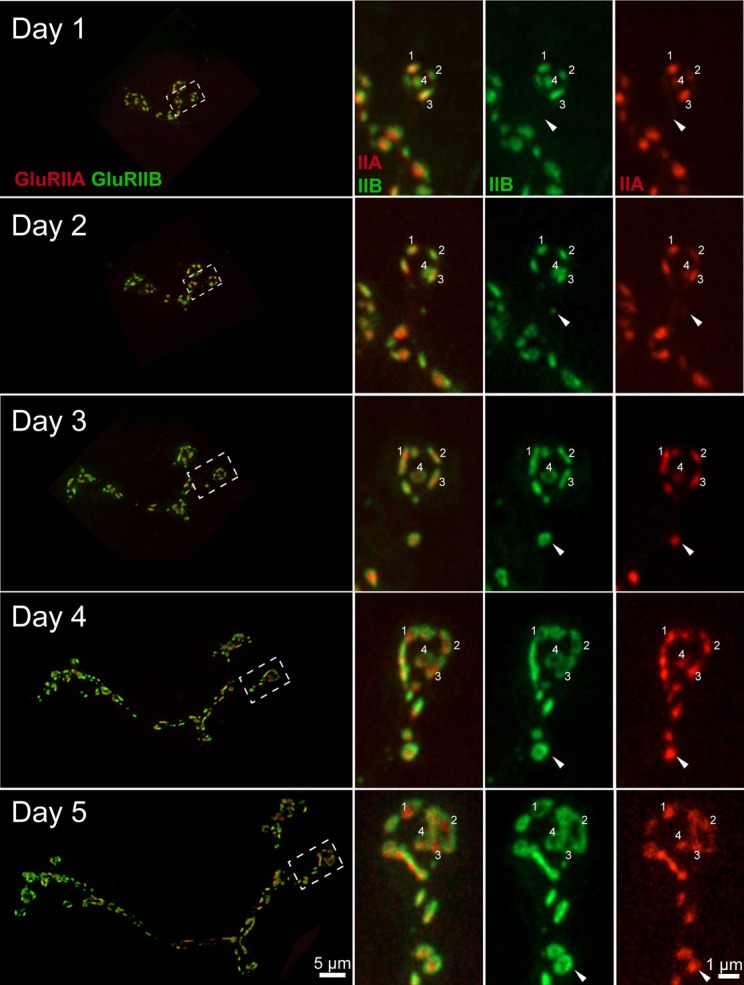
Consecutive imaging of NMJ growth at muscle 26 over a 5 day period imaged through the cuticle of an anesthetized larva during development. The entire NMJ is shown on the left panel. A smaller area (dashed box) is magnified and shown in the right panels. The merged image of GluRIIA and GluRIIB is shown in the middle, with individual GluRIIB and GluRIIA channels on the right. Several PSDs are numbered for tracking across imaging sessions. New PSDs appearing on day 2 (arrow) form the GluRIIB donut by day 4. On day 5, a larger number of PSDs display the characteristic GluRIIB peripheral segregation around a GluRIIA core, including those identified on the 1 ^st^ day of imaging (numbered).

**Figure 7—figure supplement 2.**
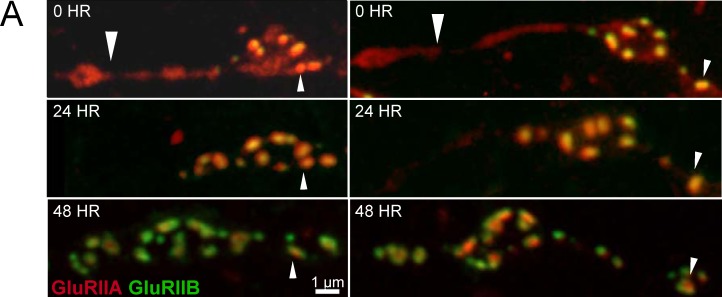
Synapse development along early GluRIIA positive NMJ extensions. (**A**) Two representative muscle 26 NMJs visualized during live imaging in early first instar larvae expressing GluRIIA-RFP and GluRIIB-GFP. The arrows denote GluRIIA-RFP positive extensions from the main arbor that are devoid of detectable PSDs or GluRIIB at this stage of development (large arrow). These extensions disappear during imaging from later larval stages, but some go on to develop fully formed synaptic boutons with many PSDs by day 2 and 3. Smaller arrows denote the same PSD at each day for orientation.

**Figure 7—figure supplement 3.**
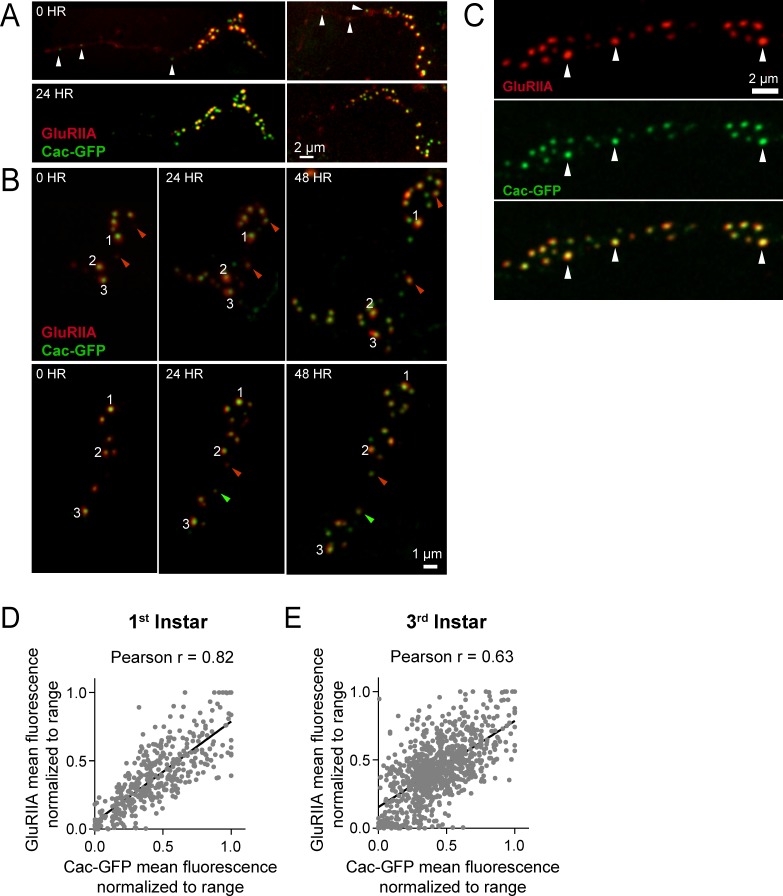
Correlation between GluRIIA-RFP and Cac-GFP fluorescence intensities throughout larval development. (**A**) Live imaging of NMJs on muscle 26 in early first instar larvae expressing GluRIIA-RFP and Cac-GFP. Long projections of GluRIIA can be seen in day 1; white arrows mark Cac-GFP puncta that have formed within these projections. (**B**) Representative Cac-GFP (green) and GluRIIA-RFP (red) synaptic puncta at a muscle 26 NMJ imaged through the cuticle of an anesthetized animal during early larval development. Red arrows mark the emergence of new AZs that first accumulate GluRIIA (day 1) and later begin to accumulate Cac-GFP and mature in size and brightness (days 2 and 3). Green arrows mark the emergence of new AZs that accumulate Cac-GFP before GluRIIA. Numbers denote AZs with bright GluRIIA and Cac-GFP on day 1. (**C**) Arrows denote AZs with bright Cac-GFP opposed to PSDs with high levels of GluRIIA. (**D, E**) Correlation between GluRIIA-RFP and Cac-GFP fluorescence at individual AZs in the first instar (**D**) and third instar (**E**) stages.

**Video 4.** Representative movie showing evoked release events in larvae expressing GluRIIA-RFP (red), GluRIIB-GFP (bright green), and postsynaptic LexAop-myrGCaMP6s.

Simultaneous expression of GluRIIA-RFP and GluRIIB-GFP revealed a heterogeneous distribution of each subunit across the AZ population, with GluRIIA levels appearing more variable than GluRIIB (Figure 7A). Similar to the relatively sparse localization of high *P_r_* AZs (Figure 1), a similar sparse distribution of AZs apposed by very bright GluRIIA fields was observed (Figure 7A). To determine if AZs that preferentially accumulate high levels of GluRIIA corresponded to high *P_r_* release sites, we mapped *P_r_* across the AZ population in GluRIIA-RFP/GluRIIB-GFP expressing animals. Analysis of the *P_r_* map revealed a strong positive correlation between mean GluRIIA-RFP intensity and *P_r_* (Pearson r = 0.56, R^2^ = 0.32, p<0.0001, n = 756 AZs from 8 NMJs from four animals; Figure 7B). In contrast, correlation with the levels of mean GluRIIB-GFP intensity was weaker (Pearson r = 0.32, R^2^ = 0.1, p<0.0001, n = 756 AZs from 8 NMJs from four animals; Figure 7C). Heterogeneity in GluRIIA PSD brightness and AZ *P_r_* was also observed in *syt1* mutants (Figure 2A, arrows). These findings are consistent with previous observations that glutamate receptors preferentially cluster at sites with high *P_r_* based on electrophysiological studies in a *Drosophila* GluRIII hypomorphic mutant (Marrus and DiAntonio, 2004b). As observed in controls (Figure 1), a positive correlation between AZ *P_r_* and BRP levels was also observed in larvae expressing labeled glutamate receptors (Pearson r = 0.44, R^2^ = 0.2, p<0.0001, n = 399 AZs from 6 NMJs from four animals; Figure 7D). In summary, these data indicate that GluRIIA accumulates more at PSDs apposing high *P_r_* AZs.

Beyond the preferential GluRIIA accumulation at high *P_r_* sites, a change in GluRIIA/B distribution within single PSDs was also observed. PSDs associated with the highest *P_r_* AZs showed a segregated distribution of the receptor subtypes, with GluRIIA concentrating in the center of the receptor field immediately apposing the presynaptic BRP cluster (Figure 7E, arrows). At these sites, GluRIIB occupied a more peripheral position around the central GluRIIA cluster. A similar localization pattern with a ring of GluRIIB surrounding a central GluRIIA patch was previously noted with antibody staining for the two receptors at a population of AZs in wildtype late third instar larvae (Marrus et al., 2004). To analyze this receptor segregation in greater detail, GluRIIA-RFP and GluRIIB-GFP were examined in the absence of co-expressed myrGCaMP6s. Prior analysis (Figure 7B) indicated the brightest GluRIIA PSDs corresponded to high *P_r_* sites. Bright PSDs were selected based on their GluRIIA intensity (two standard deviations above average) and line profiles were drawn across each PSD. The intensity of pixels along that line for each fluorophore was then analyzed. Average pixel intensity revealed drastically distinct profiles for GluRIIB distribution between ‘bright’ and ‘dim’ PSDs classified based on their GluRIIA intensity. GluRIIB was more evenly distributed across the entire PSD at dim GluRIIA sites, but was segregated outward, forming a ring around central GluRIIA puncta at bright GluRIIA sites (Figure 7F). In addition, presynaptic BRP intensity was more strongly correlated with postsynaptic GluRIIA levels (Pearson r = 0.53, R^2^ = 0.28, p<0.0001, n = 2496 AZs from 19 NMJs from seven animals; Figure 7G) than with GluRIIB (Pearson r = 0.24, R^2^ = 0.05, p<0.0001, n = 2496 AZs from 19 NMJs from seven animals; Figure 7H). These findings suggest that the postsynaptic cell accumulates GluRIIA and redistributes GluRIIB to the PSD periphery at high *P_r_* sites.

### Intravital imaging of glutamate receptors and cac channels throughout synapse development

The *Drosophila* larval NMJ is a highly dynamic structure, with new synaptic boutons and AZs undergoing continuous addition throughout development (Harris and Littleton, 2015; Rasse et al., 2005; Schuster et al., 1996; Zito et al., 1999). Given the correlation between Ca^2+^ channel abundance, GluRIIA/B segregation and high *P_r_*, we were interested in determining how AZs acquire a specific *P_r_* during a larval developmental period that lasts 6–7 days. One model is that certain AZs gain a higher *P_r_* status during development through preferential accumulation of key AZ components compared to their neighbors. Alternatively, high *P_r_* AZs might simply be more mature than their low *P_r_* neighbors, having an earlier birthdate and a longer timeframe to accumulate AZ material. To test these models for release heterogeneity, it would be desirable to follow *P_r_* development from the embryonic through larval stages. However, this is not technically feasible due to the small size of AZs and the rapid locomotion that larvae undergo, preventing generation of *P_r_* maps in moving animals. Instead, we employed an alternative intravital approach to repeatedly image the same NMJ at muscle 26 directly through the cuticle of intact larvae during anesthesia (Andlauer and Sigrist, 2012; Fouquet et al., 2009; Füger et al., 2007; Rasse et al., 2005; Zhang et al., 2010). During anesthesia, endogenous action potential-induced release and the associated GCaMP signals were eliminated, preventing direct *P_r_* measurements in anesthetized larvae. We instead focused on imaging GluRIIA accumulation and GluRIIA/B segregation, which was strongly correlated with *P_r_* (Figure 7), as a proxy for the emergence of high *P_r_* sites.

Previously described in vivo imaging approaches with anesthesia at *Drosophila* NMJs employed early third instar larvae as the starting time point, and followed the distribution of fluorescently-labeled synaptic proteins during the final ~36 hr of development prior to pupation (Andlauer and Sigrist, 2012; Fouquet et al., 2009; Füger et al., 2007; Rasse et al., 2005; Zhang et al., 2010). To follow AZ *P_r_* development beginning soon after synapse formation, we modified these techniques to allow imaging of glutamate receptors at earlier stages of development (see Materials and methods). This allowed successful birth dating and successive imaging of the same AZ over a 6 day period beginning shortly after synapse formation in the early first instar period through the late third instar stage (Figure 7—figure supplement 1). In early first instar larvae (within 12 hr of hatching) GluRIIA and GluRIIB were largely co-localized at postsynaptic puncta (Figure 7—figure supplement 1). One exception was the presence of diffuse GluRIIA that accumulated around unusually long axonal extensions that emerged from presynaptic boutons (Figure 7—figure supplement 2). These structures were devoid of any detectable GluRIIB or the bright GluRIIA puncta that are associated with AZs, and may be remnants of previously described muscle filopodial structures, termed myopodia, that interact with presynaptic filopodia to dynamically regulate early synaptic target recognition (Kohsaka and Nose, 2009; Ritzenthaler et al., 2000; Ritzenthaler and Chiba, 2003). GluRIIA was diffusely present on these structures, as has been observed for the leucine-rich repeat cell adhesion protein Capricious (Kohsaka and Nose, 2009). Repeated imaging of these thinner GluRIIA-positive processes revealed that they were capable of developing into mature synaptic boutons with concentrated GluRIIA and GluRIIB synaptic puncta (Figure 7—figure supplement 3). By 24 hr of larval growth, GluRIIA rich extensions were no longer observed, indicating these structures are restricted to early developmental stages.

Prior studies indicated GluRIIA PSD levels closely track with Cac accumulation at corresponding AZs of third instar larvae (Fouquet et al., 2009; Rasse et al., 2005), indicating the two compartments are likely to mature at similar rates. To examine this directly, we assayed whether GluRIIA and Cac accumulation were correlated during earlier stages of development (Figure 7—figure supplement 3). Indeed, the intensity of Cac-GFP and GluRIIA-RFP puncta were strongly correlated at individual AZs during both early and late larval development (first instar; Pearson r = 0.82, R^2^ = 0.6771, p<0.0001, n = 441 AZs from 8 NMJs from eight animals; third instar; Pearson r = 0.63, R^2^ = 0.395, n = 874 AZs from 8 NMJs from five animals; Figure 7—figure supplement 3D,E). One exception was observed in very early first instar larvae, where a few Cac-GFP puncta accumulated along the previously described GluRIIA positive axonal extensions prior to the specific accumulation of GluRIIA at PSDs (Figure 7—figure supplement 3A). In contrast, postsynaptic appearance of GluRIIA at the PSD could be observed to slightly precede the accumulation of Cac-GFP at later developmental stages (Figure 7—figure supplement 3B,C), similar to previous observations at mature third instar NMJs (Rasse et al., 2005). In summary, the intensity of Cac-GFP and GluRIIA-RFP puncta are strongly correlated at AZs during larval development, indicating that GluRIIA provides a robust marker that reflects the corresponding levels of presynaptic Cac at individual AZs.

### Analysis of *Pr* acquisition during AZ development using glutamate receptor segregation as a proxy

**Figure 8.**
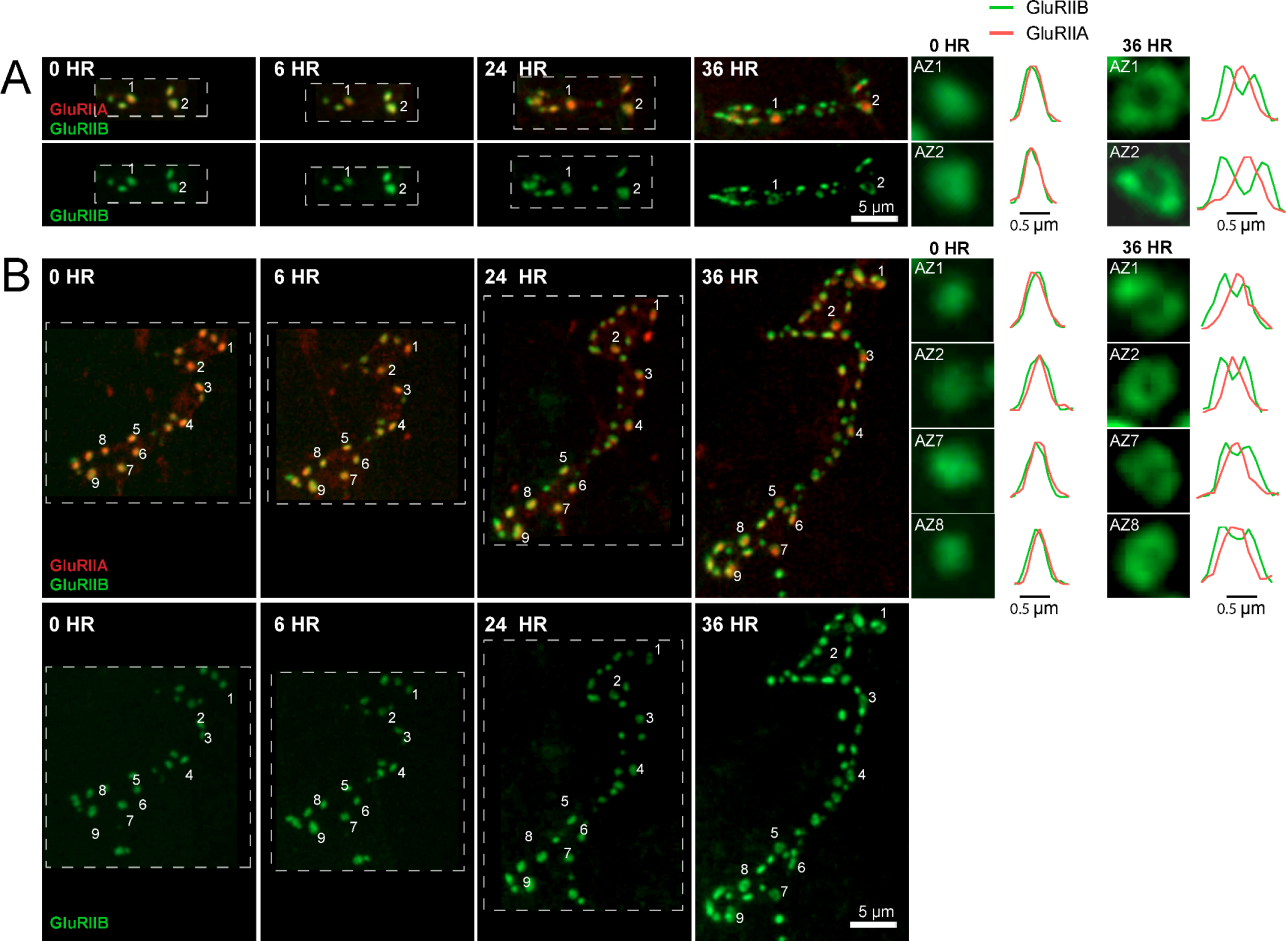
Glutamate receptor segregation during PSD development. (**A**) Representative serial time points of NMJ development visualized by repeated imaging through the cuticle of an anesthetized larvae at the indicated time points beginning at the early first instar stage. Two of the five brightest PSDs present during the first imaging session are labeled and are the first to develop the peripheral GluRIIB segregation pattern 36 hr later. GluRIIB labeling alone is shown in the bottom panel. The right panels show GluRIIB fluorescence and normalized GluRIIA and GluRIIB fluorescent line profiles for the indicated PSDs at the initial imaging session (0 hr) and 36 hr later. (**B**) Serial images of an NMJ with a larger number of AZs present at the first instar stage. After 36 hr of development, the peripheral segregation of GluRIIB around GluRIIA was first observed in some of the PSDs that were present during the initial imaging session (numbered). The right panels show GluRIIB fluorescence and normalized GluRIIA and GluRIIB fluorescent line profiles for the indicated PSDs at the initial imaging session (0 hr) and 36 hr later. The dashed box surrounds the actual imaged segment of the NMJ in each panel.

**Figure 9.**
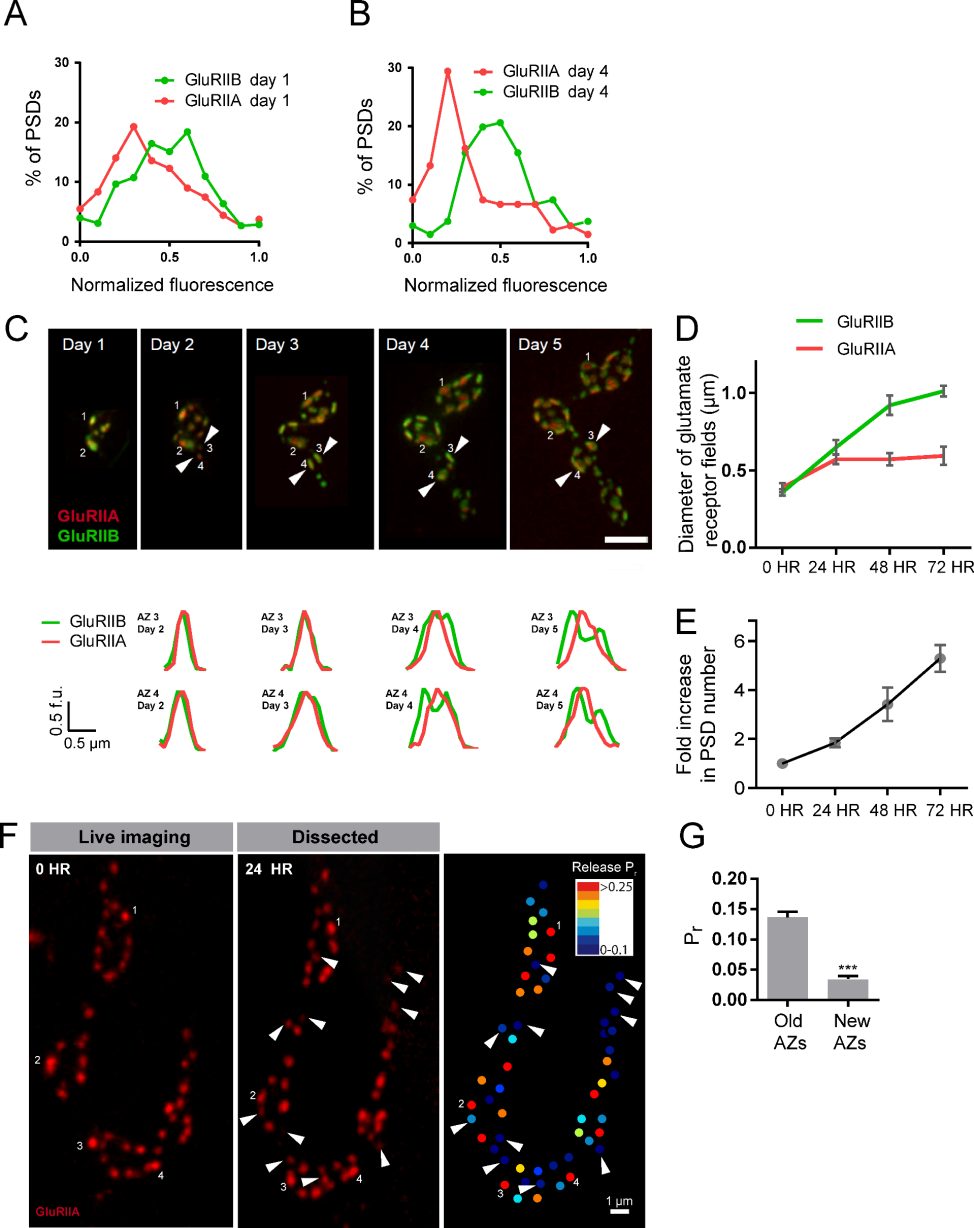
Rate of acquisition of glutamate receptor segregation during development. Histograms of the distribution of normalized GluRIIA and GluRIIB fluorescence at the first instar (day 1) (**A**) and third instar (day 4) (**B**) stages for muscle 26 imaged through the cuticle of anesthetized larvae. For each data set, GluRIIA and GluRIIB fluorescence is presented from dimmest (0) to brightest (1). GluRIIA shows a more skewed distribution of fluorescence at day 4, consistent with its accumulation at high *P_r_* AZs. (**C**) Representative muscle 26 NMJ image sequence showing appearance and maturation of two new synapses (#3 and 4) that were not present in the initial imaging session. Several preexisting synapses (#1 and 2) that developed the typical GluRIIB donut structure later in development are also labeled. The dashed box surrounds the actual imaged segment of the NMJ. New GluRIIA and GluRIIB clusters appear initially as small puncta (day 2, arrows) that become brighter on day 3. By day four they begin to display the donut like GluRIIB profile. At day 5, GluRIIB distribution to the periphery around a bright GluRIIA PSD representative of high *P_r_* sites becomes prominent. The bottom panels show normalized GluRIIA and GluRIIB fluorescent line profiles for the newly identified PSDs (#3, 4) throughout the 5 day imaging series. (**D**) Diameter of glutamate receptor fields during the first 72 hr of PSD development. Error bars represent SEM. (**E**) Changes in AZ number during larval maturation at muscle 26 presented as a ratio of AZs observed during the first day of imaging (day 1). (**F**) Representative serial time points of GluRIIA-RFP in vivo imaging over 24 hr (left two panels). Newly formed PSDs are marked with white arrows; these displayed uniformly low *P_r_* during release mapping (right panel). Several older PSDs with bright GluRIIA intensity are denoted with white numbers. (**G**) Average *P_r_* of old AZs (those present in the 0 hr time point initial imaging session) and new AZs (AZs under 24 hr old that were first seen at the 24 hr time point). Student’s t-test was used for statistical analysis (***=p ≤ 0.001). Error bars represent SEM.

We examined how GluRIIA accumulation and GluRIIA/B segregation emerged during development of the NMJ. Live imaging of GluRIIA and GluRIIB distribution at early PSDs in anesthetized first instar larvae demonstrated that the receptors were co-localized and lacked the segregation where GluRIIB clustered around central GluRIIA puncta that was observed at high *P_r_* sites later in development (Figure 8A). The first emergence of GluRIIA/B segregation was observed after 36 hr of imaging starting from the first instar period. The GluRIIA/B segregation always emerged first at the oldest synapses that existed previously on the first day of imaging (Figure 8A,B). The most mature PSDs also contained more GluRIIA fluorescent signal (17430 ± 634.0, n = 86 AZs from 8 NMJs from five animals) compared to younger synapses that emerged during the 48 hr imaging session (8909 ± 289.8, n = 210 AZs from 8 NMJs from five animals). During later larval development, the cuticle thickness changed dramatically and prevented reliable comparison of absolute receptor density with earlier stages. However, GluRIIA intensities that were more uniform at the first instar larval stage became more heterogeneous at the third instar stage (Figure 8—figure supplement 1). Indeed, histograms of normalized fluorescence intensity (relative intensity scaled from 0 to 1) revealed that GluRIIA and GluRIIB were relatively uniformly distributed at first instar larval PSDs, with GluRIIA distribution becoming more skewed at later stages (Figure 9A,B). To determine whether muscle 26 exhibits *P_r_* heterogeneity similar to muscle 4, we performed *P_r_* mapping using GluRIIA-RFP and myr-GCaMP6s in dissected non-anesthetized third larvae and confirmed that *P_r_* heterogeneity is extremely similar between muscle 26 (mean *P_r_* = 0.068 ± 0.0048, skewness = 2.27, 365 AZs from 5 NMJs from five animals) and muscle 4 (mean *P_r_* = 0.073 ± 0.0021, skewness = 2.23, 1933 AZs from 16 NMJs from 16 animals; Figure 9—figure supplement 1B–D). These results indicate that GluRIIA/B fluorescence and *P_r_* distribution are both highly heterogeneous by the early third instar stage in muscle 26, with the brightest GluRIIA PSDs, and by extension their corresponding high *P_r_* AZs, representing those that appeared earliest in development.

**Figure 8—figure supplement 1.**
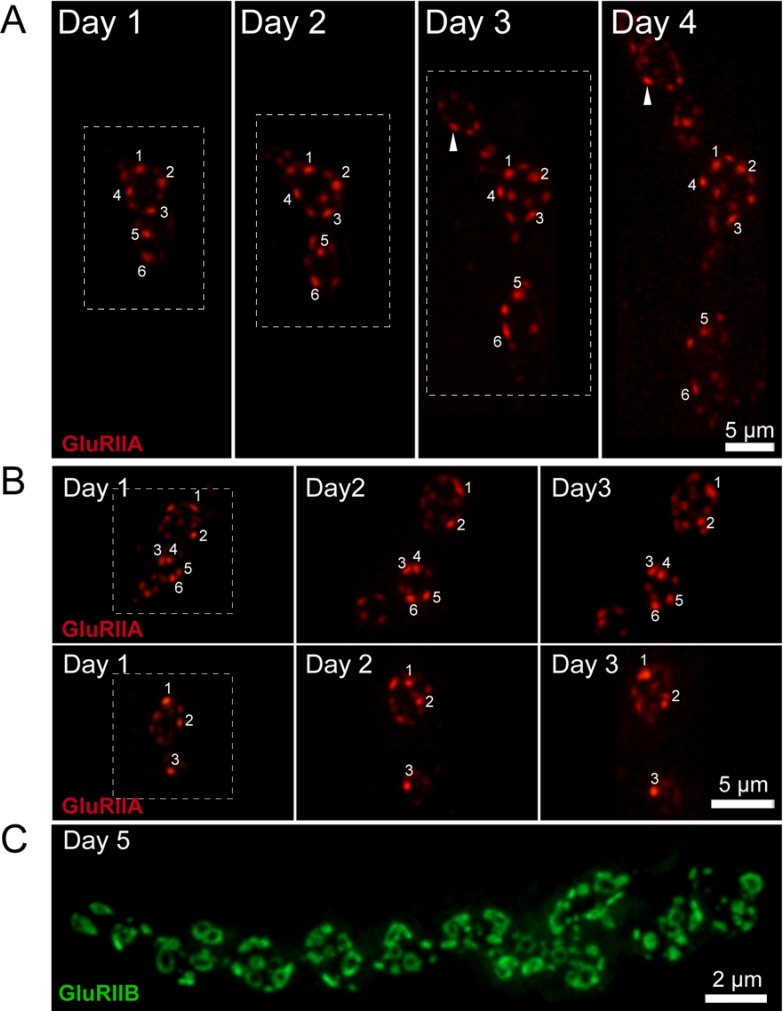
GluRIIA fluorescence intensity increases over time in a rate proportional to PSD birthdate. Representative serial images of muscle 26 NMJs visualized through the cuticle of two anesthetized larvae during development (**A, B**). The dashed box surrounds the actual imaged segment of the NMJ in each panel. The brightest GluRIIA puncta are numbered and followed through the imaging period. The brightest GluRIIA puncta observed on day one were among the brightest puncta on later days. Rarely, formation of new PSDs that showed a faster rate of GluRIIA accumulation were observed (arrow). (**C**) By day 5 of larval development, many PSDs show the donut-like GluRIIB distribution, though the number of smaller GluRIIB puncta also increase due to new AZ addition.

**Figure 9—figure supplement 1.**
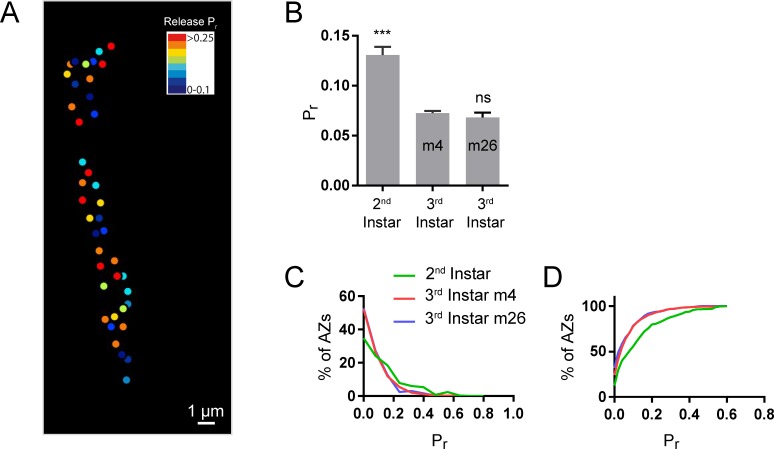
Comparison of evoked *P_r_* at muscle 4 and muscle 26. (**A**) Representative evoked *P_r_* map in second instar larvae, muscle 4. (**B**) Average evoked *P_r_* for second instar muscle 4, third instar muscle 4, and third instar muscle 26. Student’s t-test was used for statistical analysis (***=p ≤ 0.001). Error bars represent SEM. (**C**) Histogram and (**D**) cumulative frequency distribution of evoked *P_r_* in second instar muscle 4, third instar muscle 4, and third instar muscle 26.

Over what time frame do synapses developmentally acquire markers of high *P_r_* sites? To estimate the average time required for AZ maturation, we calculated the time interval from the first emergence of a PSD in an imaging session to the time point when segregation of GluRIIB around GluRIIA central puncta occurred. This analysis was restricted to newly formed AZs that appeared during the imaging sessions and excluded AZs that were present in the first imaging session performed in first instar larvae. The average time from the first emergence of a PSD to when it acquired the segregated GluRIIA/B pattern observed at high *P_r_* AZs was 3.20 ± 0.08 days (n = 41 AZs from 7 NMJs from three animals; Figure 9C). In a small subset of PSDs (5%), a slightly faster accumulation of GluRIIA and the formation of GluRIIB peripheral rings was observed, but never faster than 2 days. In addition, glutamate receptor fields increased in size throughout the first 72 hr of development (Figure 9D). GluRIIA diameter increased by 1.47-fold during the first 24 hr of development but then plateaued, while GluRIIB field diameter continued to grow over 72 hr, increasing in size by 2.8-fold over that time. At 72 hr after initial glutamate receptor field formation, the average GluRIIA field diameter was 0.59 ± 0.058 μm (range 0.45–0.80 μm, 12 PSDs from three animals) and the average GluRIIB field diameter was 1.01 ± 0.035 μm (range 0.89–1.12 μm, 12 PSDs from three animals), consistent with the formation of segregated GluRIIA/B fields at mature AZs.

To directly assess whether AZ age corresponds to *P_r_*, we followed PSDs in animals expressing GluRIIA-RFP, GluRIIB-GFP and myr-GCaMP for 24 hr using in vivo imaging, and then dissected the animals and mapped *P_r_* directly (Figure 9F). We observed that PSDs that emerged on the second day of imaging (less than 24 hr old) were consistently associated with very low *P_r_* AZs. The mean *P_r_* of these newly-formed AZs was 0.035 ± 0.0047 (n = 77 AZs from 4 NMJs from four animals), with a range of 0–0.14. In contrast, the average *P_r_* for AZs older than 24 hr was 0.14 ± 0.0093 with a range of 0–0.61 (n = 188 AZs from 4 NMJs from four animals; Figure 9G). These findings support a model in which the vast majority of newly formed AZs are very weak, with increased *P_r_* requiring more than 24 hr to develop.

Given the developing NMJ is adding AZs at a rapid rate (Rasse et al., 2005; Schuster et al., 1996), we estimated whether AZ maturation time identified over the course of our live imaging experiments could lead to the ~10% of high *P_r_* sites observed at the early third instar stage. We quantified the number of synapses present at the same NMJ from the first instar through the early third instar stage from live imaging experiments (Figure 9E). AZ number roughly doubled each day, such that the average number of AZs found at the first instar stage (day 1) represented 14.7 ± 1.4% (n = 8 NMJs from three animals) of all AZs present by day 4 (3 days after initial imaging in first instars). Overall, these data are consistent with the hypothesis that AZ maturation is a key factor in regulating *P_r_*, leading to increased accumulation of Ca^2+^ channels and GluRIIA/B segregation at high *P_r_* sites compared to AZs that are newly formed (<2 days).

If heterogeneity in AZ age underlies the majority of diversity in *P_r_*, we would expect to see a different *P_r_* distribution at the earlier second instar stage when there is a smaller range of AZ ages. Since the number of AZs nearly doubles each day and newly formed AZs display low *P_r_*, we hypothesized that third instar NMJs would have a greater proportion of low *P_r_* sites compared to second instar NMJs. To test this hypothesis, we mapped *P_r_* at muscle 4 NMJs in the second instar and compared the distribution to that seen at the third instar stage (Figure 9—figure supplement 1A,B). A rightward shift in the distribution of *P_r_* was observed in the earlier second instar stage, with a greater proportion of AZs in the higher *P_r_* category, a smaller population of low *P_r_* AZs, and a significant increase in mean *P_r_* in second instars compared to third instars (third instar: 0.07 ± 0.002, n = 1933 AZs from 16 NMJs from 16 animals; second instar: 0.13 ± 0.008, n = 282 AZs from 6 NMJs from six animals; Figure 9—figure supplement 1C,D). We were unable to map *P_r_* at muscle 26 in earlier stages in dissected animals because it is covered by muscles 6 and 7 until the third instar stage. These data support the hypothesis that *P_r_* heterogeneity reflects AZ age and maturation time.

### Postsynaptic maturation rate depends on presynaptic activity

**Figure 10.**
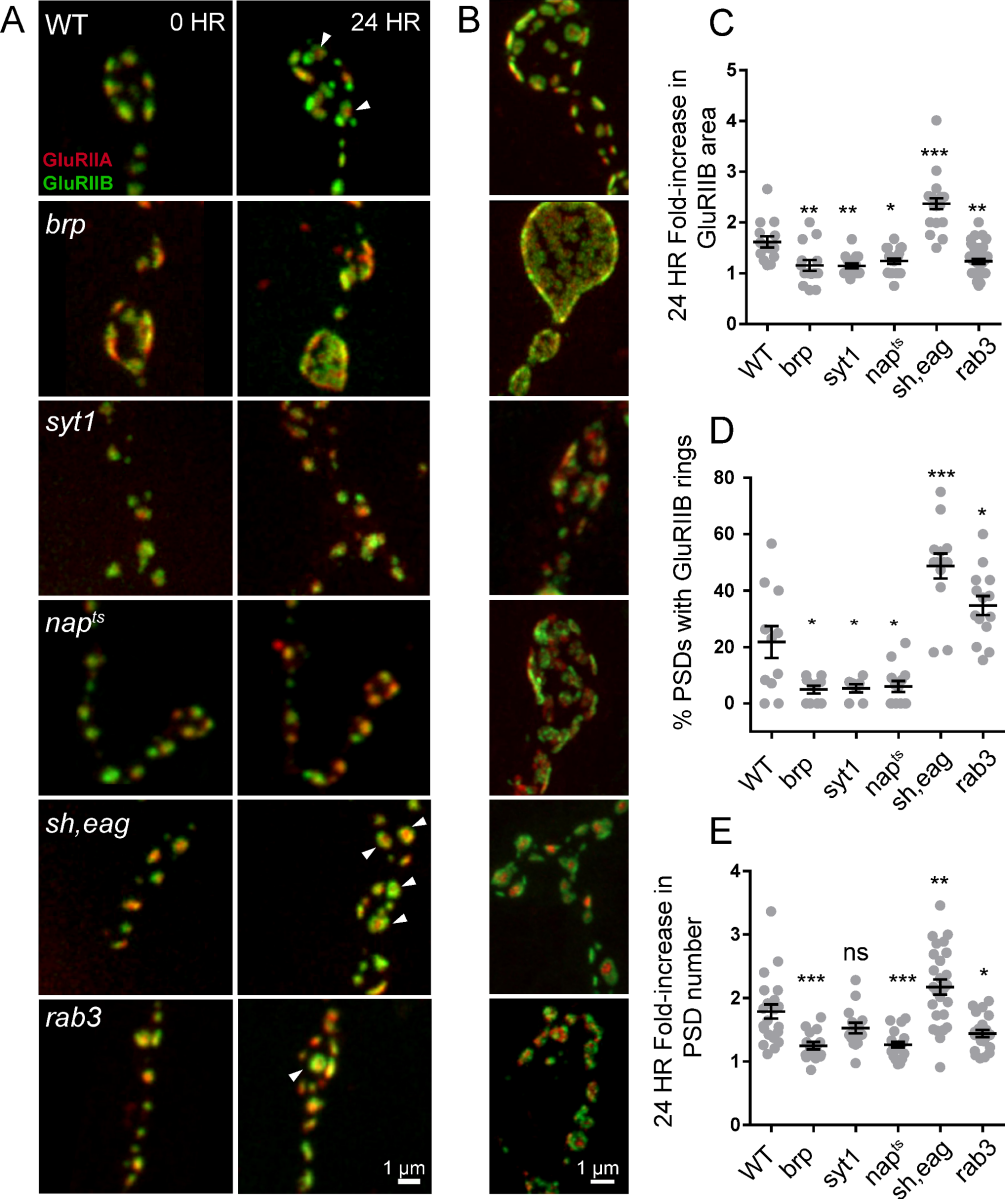
Glutamate receptor field maturation in mutants with altered presynaptic activity. (**A**) Representative NMJs on muscle 26 for each genotype imaged intravitally over 24 hr; the 0 hr timepoint (left column) corresponds to imaging during the early first instar stage. White arrows (24 hr) denote PSDs that have acquired GluRIIA/IIB segregation at this time point. (**B**) Representative third instar PSDs. (**C**) Fold-increase in GluRIIB area over 24 hr in newly formed PSDs. (**D**) Percent of PSD fields with GluRIIB rings at the second instar stage. A PSD was considered to have a GluRIIB ring if the line profile through the GluRIIB field had a central dip in fluorescence intensity of greater than 10%. (**E**) Fold increase in PSD number, defined by the number of GluRIIB puncta, over 24 hr of imaging beginning in the first instar stage. (**C–E**) Each point represents the average from one NMJ. One way ANOVA followed by Dunnett’s multiple comparisons test was used for statistical analysis (*=p ≤ 0.05, **=p ≤ 0.01, ***=p ≤ 0.001). Error bars represent SEM.

To determine whether the developmental time-course of synapse maturation could be modulated by changes in presynaptic activity, we measured the rate of PSD growth (fold-increase in GluRIIB area over 24 hr) and the percent of PSDs displaying GluRIIA/B segregation in mutants with altered presynaptic activity (Figure 10). We first measured postsynaptic maturation in *BRP^69/def^* null animals, which have a dramatic reduction in evoked synaptic transmission (Kittel et al., 2006). Consistent with previous findings, Cac-GFP intensity in *BRP^69/def^* animals was reduced to 25% (mean fluorescence intensity 3549 ± 23, 4 NMJs from two animals) of control levels (mean intensity 14177 ± 220, 4 NMJs from four animals; Figure 10—figure supplement 1A,B). We expressed GluRIIA-RFP and GluRIIB-GFP in *BRP^69/def^* mutants and imaged muscle 26 NMJs in anesthetized larvae intravitally over 24 hr. A significant reduction in postsynaptic maturation rate was observed; newly formed *BRP^69/def^* PSDs only increased in GluRIIB area by 1.16-fold (±0.11, 4 NMJs from three animals) over the first 24 hr of development, compared to a 1.61-fold (±0.11, 5 NMJs from five animals) increase in controls (Figure 10A,C). Furthermore, a significant reduction in the percent of PSDs with GluRIIA/B rings (defined by a > 10% central dip in the GluRIIB intensity profile) was observed in the second day of larval development; only 4.9% of *BRP^69/def^* PSDs (±1.3%, 10 NMJs from five animals) showed receptor segregation compared to 22% (±5%, 11 NMJs from five animals) in age-matched controls (Figure 10D). We were unable to observe any GluRIIB rings at the third instar stage in *BRP^69/def^*, in contrast to the clear rings seen in controls at this stage. Instead, *BRP^69/def^* mutants displayed highly disorganized GluR clusters (Figure 10B).

**Figure 10—figure supplement 1.**
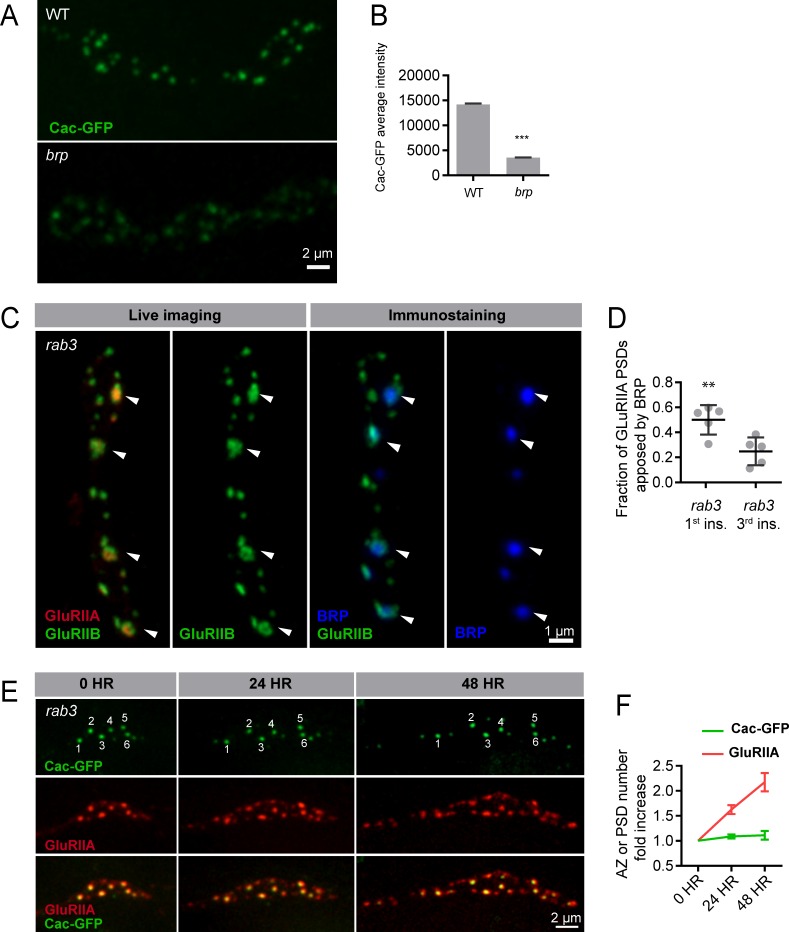
Synapse development in *Rab3* mutants. (**A**) Representative images of UAS-Cac-GFP driven in neurons using elav-Gal4 in control and *brp^69/def^* backgrounds. (**B**) Cac-GFP average intensity per AZ in control and *brp^69/def^* backgrounds. (**C**) Muscle 26 of *rab3* first instar animals expressing GluRIIA-RFP and GluRIIB-GFP imaged live (left two panels) and then fixed and immunostained for BRP (blue; right two panels). Arrows denote release sites with mature glutamate receptor fields; these sites are enriched in BRP. (**D**) Fraction of GluRIIA PSDs apposed by AZs containing BRP in first instar vs. third instar *rab3* larvae. (**E**) *Rab3* animals expressing UAS-Cac-GFP and GluRIIA-RFP followed intravitally over 48 hr of development, beginning in the first instar stage (day 1). Numbers (top row) mark AZs enriched in Cac-GFP on day 1, showing these AZs remain stably enriched for Cac-GFP throughout the 48 hr imaged. (**F**) Fold increase in number of GluRIIA puncta (red) and Cac-GFP puncta (green) every 24 hr. Student’s t-test was used for statistical analysis (***=p ≤ 0.001). Error bars represent SEM.

To examine the consequences of reductions in presynaptic activity independent of structural alterations in the AZ that might occur at NMJs in *BRP* mutants, we measured PSD maturation rate in *nap^TS^* and *syt1^null^* mutants that reduce release through separate mechanisms. Loss of Syt1 causes a dramatic decrease in synchronous fusion (DiAntonio and Schwarz, 1994; Guan et al., 2017; Lee et al., 2013; Littleton et al., 1994, 1993; Yoshihara et al., 2003; Yoshihara and Littleton, 2002), while *nap^TS^* results in constitutively reduced neuronal excitability due to decreased sodium channel activity (Kernan et al., 1991; Wu et al., 1978). In both *syt1* and *nap^TS^* mutants, significantly reduced PSD growth rate and GluRIIA/B segregation was observed compared to controls; GluRIIB field area in *nap^TS^* and *syt1^null^* mutants only increased by 1.24-fold (±0.05 4 NMJs from three animals) and 1.15-fold (±0.05, 4 NMJs from four animals) respectively during the first 24 hr of PSD development compared to 1.61-fold (±0.11, 5 NMJs from five animals) in controls (Figure 10C). At the second instar stage, when 22% of control PSDs displayed GluRIIA/B segregation, only 5.4% of *syt1* (±1.5%, 7 NMJs from three animals) and 6.0% of *nap^TS^* (±2.0%, 13 NMJs from four animals) PSDs showed GluR segregation (Figure 10D). Both *nap^TS^* and *syt1^null^* mutants also formed less segregated GluRIIA/B fields compared to controls at the third instar stage (Figure 10B). To determine whether increasing presynaptic release would accelerate maturation, we examined PSD size and GluR segregation in *shaker^120^, eag^1^* (*sh, eag*) double mutants that display increased excitability due to loss of several K^+^ currents (Ganetzky and Wu, 1983; Salkoff and Wyman, 1981; Wu et al., 1983). *Sh, eag* showed a significantly increased rate of GluRIIB field size increase (2.37-fold ± 0.11, 5 NMJs from four animals) over 24 hr compared to controls (1.61 fold (±0.11, 5 NMJs from five animals; Figure 10A,C). Furthermore, 48.7% of *sh*, *eag* mutant PSDs (±4.4%, 13 NMJs from five animals) displayed GluRIIB rings at the early second instar stage, compared to only 22% of control PSDs (Figure 10D).

These observations indicate that increasing (*sh, eag*) or decreasing (*nap^TS^*, *BRP^69/def^, syt1*) presynaptic activity results in a respective increase or decrease in PSD maturation rate. One factor that might contribute to these changes in PSD maturation rate is altered growth rate of the entire NMJ secondary to changes in neuronal activity. Indeed, we observed that the rate of new PSD addition over 24 hr in *BRP^69/def^, nap^TS^,* and *sh, eag* mutants showed a similar trend to PSD maturation, suggesting that overall NMJ growth rate is altered in these mutants (control animals increase AZ number by 1.8 ± 0.1 fold over 24 hr compared to *sh, eag* (2.2 ± 0.1 fold), *nap^TS^* (1.3 ± 0.04 fold), *syt1* (1.5 ± 0.08 fold), and *BRP^69/def^* (1.2 ± 0.06 fold); Figure 10E). Previous work on activity-dependent NMJ growth in *Drosophila* focused primarily on bouton number, not AZ addition rate, and it will be interesting to explore the molecular mechanisms mediating activity-dependent seeding of new AZs. To measure PSD maturation rate in the context of an otherwise healthy animal, we took advantage of the *rab3* null mutant, where roughly half of the PSDs are apposed by presynaptic areas lacking BRP and Cac, and the remaining AZs contain higher than normal levels of these presynaptic components (Graf et al., 2009). *Rab3* AZs containing BRP and Cac have been shown to have higher *P_r_*, whereas AZs lacking these presynaptic components are functionally silent (Peled and Isacoff, 2011). This redistribution of *P_r_* provides an opportunity to compare PSD maturation with less influence of overall animal health and NMJ growth rate.

The *rab3* phenotype has been previously analyzed at the third instar stage of larval development. To characterize the dynamics of pre- and postsynaptic maturation during earlier development, we followed *rab3* animals expressing Cac-GFP and GluRIIA-RFP for 48 hr starting in the early first instar larval stage. AZs populated with Cac-GFP early in development were stable over time; Cac-GFP was never lost at these AZs over 48 hr of imaging (Figure 10—figure supplement 1E). However, a divergence in the rate of PSD versus Cac-GFP addition was observed after the first instar stage; new GluRIIA-containing PSDs were added to the NMJ at a rate similar to control, with total PSD number nearly doubling every 24 hr. However, the rate of addition of new Cac-GFP-containing AZs was much lower than in controls, resulting in a decrease in Cac/GluRIIA apposition in *rab3* mutants over development (Figure 10—figure supplement 1D,F). This is consistent with the observation that PSD growth rate was decreased in the *rab3* null (1.23-fold ± 0.05, 6 NMJs from six animals) when we measured PSDs born in the late first instar or early second instar stage (Figure 10C), while the percent of GluRIIA/B rings seen in the second instar stage was elevated (34.7 ± 3.3% of PSDs had rings, 14 NMJs from six animals) compared to age matched controls (22%; Figure 10D). To avoid complications from the atypical dynamics of AZ apposition during *rab3* NMJ development, we focused the remainder of our analysis on the early first instar stage where the age distribution of AZs is much narrower.

We next examined first instar *rab3* mutants to determine whether AZs with higher presynaptic activity had more mature postsynaptic receptor fields than their silent neighbors. We imaged GluRIIA-RFP and GluRIIB-GFP in *rab3* 1^st^ instars and then dissected and stained for BRP. In muscle 26 of *rab3* mutants, roughly 50% of first instar AZs were populated with BRP (the ratio of GluRIIA-RFP puncta to Cac-GFP puncta was 1.9 ± 0.15); these AZs were opposed by large PSDs, many of which already showed GluRIIA/IIB segregation (Figure 10D, Figure 10—figure supplement 1C). In contrast, PSDs lacking BRP were much smaller and lacked receptor segregation. All of the PSDs with GluRIIB rings were BRP-positive, suggesting that PSDs opposite AZs with reduced presynaptic release are less mature at this stage of larval development. Though the mechanism by which rab3 regulates AZ assembly remains unknown, the larger PSD size and enhanced GluRIIA/B segregation opposite BRP-positive AZs and the small size and lack of receptor segregation opposite BRP-negative AZs within the same terminal suggests that PSD maturation depends on presynaptic activity at the level of individual AZs.

## Discussion

In the current study we used quantal imaging, super resolution SIM, and intravital imaging to examine the development of heterogeneity in evoked *P_r_* across the AZ population at *Drosophila* NMJs. We first confirmed that release heterogeneity was not caused by summation of fusion events from multiple unresolvable AZs. Indeed, high *P_r_* sites corresponded to single AZs with enhanced levels of BRP. These findings are consistent with previous observations using conventional light microscopy that indicate *P_r_* correlates with BRP levels (Muhammad et al., 2015; Peled et al., 2014; Reddy-Alla et al., 2017). By monitoring release over intervals of extensive vesicle fusion during strong stimulation, we also observed that *P_r_* is a stable feature of each AZ. In addition, loss of the synaptic vesicle Ca^2+^ sensor Syt1 globally reduced *P_r_* without altering the heterogeneous distribution of *P_r_* across AZs, indicating that AZ-local synaptic vesicle pools with differential Ca^2+^ sensitivity are not likely to account for *P_r_* heterogeneity. Since VGCC abundance, gating, and organization within the AZ are well established regulators of *P_r_* across synapses (Borst and Sakmann, 1996; Chen et al., 2015; [Bibr bib72][Bibr bib124]; Nakamura et al., 2015; Sheng et al., 2012; Südhof, 2012; Wang et al., 2008), heterogeneity in presynaptic Ca^2+^ channel abundance was a clear candidate for the generation of *P_r_* heterogeneity at *Drosophila* NMJs. Indeed, the Cac Ca^2+^ channel responsible for neurotransmitter release is heterogeneously distributed across the NMJ (Fouquet et al., 2009; Kawasaki et al., 2004; 2000; Littleton and Ganetzky, 2000; Liu et al., 2011; Rieckhof et al., 2003; Smith et al., 1996). Using transgenically labeled Cac lines, we observed that Cac density at AZs is indeed strongly correlated with *P_r_*. To directly visualize presynaptic Ca^2+^ influx at single AZs, we generated GCaMP fusions to the core AZ component BRP. Ca^2+^ influx at single AZs was highly correlated with both Cac density and *P_r_*. The *cac^NT27^* mutant with decreased conductance also resulted in a global reduction in *P_r_* without disrupting heterogeneity, further confirming that Ca^2+^ influx regulates *P_r_* across the range of release heterogeneity.

Postsynaptically, high *P_r_* AZs were enriched in GluRIIA-containing receptors and displayed a distinct pattern of glutamate receptor clustering. While most synapses showed GluRIIA and GluRIIB spread over the entire PSD, high *P_r_* AZs were apposed by PSDs where GluRIIA concentrated at the center of the AZ, with GluRIIB forming a ring at the PSD periphery. Indeed, anti-glutamate receptor antibody staining of wildtype larvae lacking tagged glutamate receptors had previously identified a GluRIIB ring around the GluRIIA core in some mature third instar NMJ AZs (Marrus et al., 2004). In addition, activity-dependent segregation of GluRIIA and a GluRIIA gating mutant has been observed at individual AZs in *Drosophila* (Petzoldt et al., 2014). The correlation of *P_r_* with GluRIIA accumulation is especially intriguing considering that this subunit has been implicated in homeostatic and activity-dependent plasticity (Davis, 2006; Frank, 2014; Petersen et al., 1997; Sigrist et al., 2003). By following the developmental acquisition of this postsynaptic property as a proxy for *P_r_* from the first through third instar larval stages via intravital imaging in control and mutant backgrounds, we observed that the earliest formed AZs are the first to acquire this high *P_r_* signature over a time course of ~3 days, and that PSD maturation rate can be modulated by changes in presynaptic activity.

Similar to prior observations (Melom et al., 2013; Peled and Isacoff, 2011), we found that most AZs at the *Drosophila* NMJ have a low *P_r_*. For the current study, the AZ pool was artificially segregated into low and high release sites, with high releasing sites defined based on having a release rate greater than two standard deviations above the mean. Given that birthdate is a key predictor of glutamate receptor segregation, and by proxy *P_r_*, we expect the AZ pool to actually reflect a continuum of *P_r_* values based on their developmental history. However, using the two standard deviation criteria, 9.9% of AZs fell into the high *P_r_* category, with an average *P_r_* of 0.28. We also observed that 9.7% of the AZs analyzed displayed only spontaneous release. We could detect no fusion events for either evoked or spontaneous release for another 14.6% of AZs that were defined by a GluRIIA-positive PSD in live imaging. Future investigation will be required to determine whether these cases represent immature AZs with extremely low evoked *P_r_*, or distinct categories reflective of differences in AZ content. The remaining AZs that participated in evoked release had an average *P_r_* of 0.05. Ca^2+^ channel density and Ca^2+^ influx at individual AZs was a key determinant of evoked *P_r_* heterogeneity, as *P_r_* and the intensity of Cac channels tagged with either TdTomato or GFP displayed a strong positive correlation. Spontaneous fusion showed a much weaker correlation with both Cac density and Ca^2+^ influx at individual AZs, consistent with prior studies indicating spontaneous release rates are poorly correlated with external Ca^2+^ levels at this synapse (Jorquera et al., 2012; Lee et al., 2013). With synaptic vesicle fusion showing a steep non-linear dependence upon external Ca^2+^ with a slope of ~3–4 (Dodge and Rahamimoff, 1967; Heidelberger et al., 1994; Jan and Jan, 1976), a robust change in *P_r_* could occur secondary to a relatively modest increase in Ca^2+^ channel abundance over development. Although the number of VGCCs at a *Drosophila* NMJ AZ is unknown, estimates of Cac-GFP fluorescence during quantal imaging indicate a ~ 2 fold increase in channel number would be necessary to move a low *P_r_* AZ into the high *P_r_* category. Similar correlations between evoked *P_r_* and Ca^2+^ channel abundance have been found at mammalian synapses (Holderith et al., 2012; Nakamura et al., 2015; Sheng et al., 2012), suggesting this represents a common evolutionarily conserved mechanism for determining release strength at synapses.

We did not test the correlation of *P_r_* with other AZ proteins besides Cac and BRP, but it would not be surprising to see a positive correlation with the abundance of many AZ proteins based on the observation that maturation time is a key determinant for *P_r_*. Indeed, recent studies have begun to correlate *P_r_* with specific AZ proteins at *Drosophila* NMJs (Reddy-Alla et al., 2017). We also observed that PSD size was robustly increased by 1.6-fold over a 24 hr period of AZ development during the early larval period in control animals. AZ maturation is likely to promote increased synaptic vesicle docking and availability, consistent with observations that correlate AZ size with either *P_r_* or the readily releasable pool (Han et al., 2011; Holderith et al., 2012; Matkovic et al., 2013; Matz et al., 2010; Nakamura et al., 2015; Schikorski and Stevens, 1997; Wadel et al., 2007).

We considered several models for how AZs acquire this heterogeneous nature of *P_r_* distribution during a developmental period lasting several days. One possibility is that unique AZs gain high *P_r_* status through a mechanism that would result in preferential accumulation of key AZ components compared to their neighbors. Given that retrograde signaling from the muscle is known to drive synaptic development at *Drosophila* NMJs (Ball et al., 2010; Berke et al., 2013; Harris and Littleton, 2015; Keshishian and Kim, 2004; McCabe et al., 2003; Piccioli and Littleton, 2014; Yoshihara et al., 2005), certain AZ populations might have preferential access to specific signaling factors that would alter their *P_r_* state. Another model is that AZs compete for key presynaptic *P_r_* regulators through an activity-dependent process. High *P_r_* AZs might also be more mature than their low *P_r_* neighbors, having a longer timeframe to accumulate AZ components. Given the *Drosophila* NMJ is constantly forming new AZs at a rapid pace during development (Rasse et al., 2005; Schuster et al., 1996), newly formed AZs would be less mature compared to a smaller population of ‘older’ high *P_r_* AZs.

**Figure 11.**
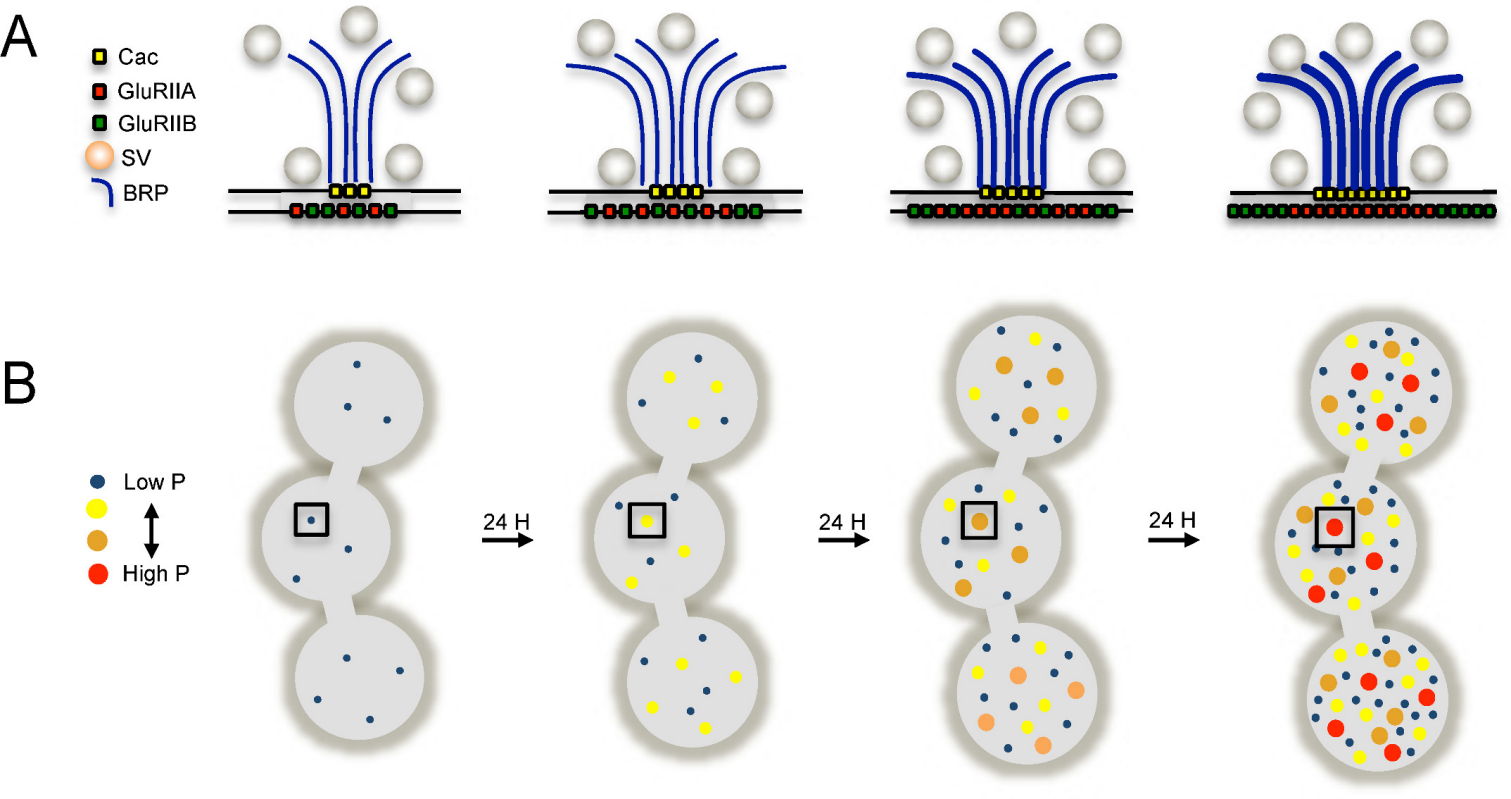
Model depicting the acquisition of release heterogeneity over three days of NMJ development. (**A**) Presynaptically, Ca^2+^ channels (yellow) and BRP (blue) are added over time to increase release probability. Postsynaptically, GluRIIA and GluRIIB are added during maturation and eventually segregate at high releasing sites. (**B**) The number of AZs roughly doubles every 24 hr, with all new AZs having low release probability (blue dot). AZs increase in release probability over time and eventually reach high *P_r_* status (red dot).

To examine if the release heterogeneity observed at the third instar stage reflects AZ birth order over several days of development, we needed to extend the intravital imaging through a longer time period beginning in the first instar larval stage. GCaMP imaging indicated high *P_r_* sites segregate GluRIIA and GluRIIB differently from low *P_r_* sites, with the IIA isoform preferentially localizing at the center of PSDs apposing high *P_r_* AZs. As such, we used developmental acquisition of this property as an indicator of high *P_r_* sites. Although segregation of glutamate receptors may not perfectly replicate the timing of *P_r_* acquisition during development, it is currently the best tool for estimating *P_r_* during sequential live imaging. Based on the acquisition of GluRIIA/B segregation, the data support the hypothesis that increases in *P_r_* reflect a time-dependent maturation process at the NMJ. The continuous addition of new AZs, which double in number during each day of development, ensures that the overall ratio of high to low *P_r_* sites represents a low percentage as the NMJ grows (Figure 11). We further established confidence in the age-dependency model of *P_r_* by mapping release after following NMJs intravitally for 24 hr; using this approach, we found that newly formed AZs are consistently very low *P*_r_. Finally, we mapped *P_r_* in the second instar stage and observed that the heterogeneity at this stage is shifted towards higher releasing sites, with a reduction in the fraction of low-releasing sites.

These data indicate AZs are born with low *P_r_* and gain pre- and postsynaptic material over 3 days on an upward trajectory toward higher *P_r_* status (Figure 11). Is AZ age a static determinant of *P_r_*, or can growth rate be regulated to allow faster or slower acquisition of high *P_r_* AZs? To answer this question, we assayed whether mutants that alter presynaptic activity influence the rate of PSD growth and GluRIIA/B segregation. In *BRP^69/def^*, *syt1^null^,* and *nap^TS^* mutants with decreased presynaptic release, a significant reduction in postsynaptic maturation rate and in the percentage of PSDs with GluRIIA/IIB rings was observed. Conversely, in the *shaker, eag* double mutant with increased presynaptic excitability, a significantly increased rate of GluRIIB field size and a significant increase in GluRIIA/IIB rings was found compared to controls. To investigate whether differences in synapse growth and maturation rate could be seen between AZs enriched in BRP and Cac versus neighboring AZs that are deficient in these components, we imaged development in the *rab3* null mutant. At the first instar stage when AZs are roughly age matched, release sites enriched in presynaptic components had developed large and mature PSDs in stark contrast with non-enriched AZs, whose PSDs appeared immature. These results indicate that PSD maturation can be influenced by presynaptic activity at the resolution of single AZs.

Although how AZs are assembled during development is still being established, our data do not support a model where AZs are fully preassembled during transport and then deposited as a single ‘quantal’ entity onto the presynaptic membrane. Rather, these data support a model of seeding of AZ material that increases developmentally over time as AZs matures, consistent with previous studies of AZ development in *Drosophila* (Böhme et al., 2016; Fouquet et al., 2009). Although no evidence for rapid changes in *P_r_* were detected in the steady-state conditions used in the current study, homeostatic plasticity is known to alter *P_r_* over a rapid time frame (~10 min) at the NMJ (Davis and Müller, 2015; Frank, 2014; Frank et al., 2006). It will be interesting to determine if Cac abundance can change over such a rapid window, or whether the enhanced release is mediated solely through changes in Cac function and Ca^2+^ influx (Müller and Davis, 2012). Changes in the temporal order of *P_r_* development could also occur secondary to altered transport or capture of AZ material. For example, the large NMJ on muscle fibers 6 and 7 displays a gradient in synaptic transmission, with terminal branch boutons often showing a larger population of higher *P_r_* AZs (Guerrero et al., 2005; Peled and Isacoff, 2011). If AZ material is not captured by earlier synapses along the arbor, it would be predicted to accumulate in terminal boutons, potentially allowing these AZs greater access to key components, and subsequently increasing their rate of *P_r_* acquisition. Alternatively, the gradient of *P_r_* along the axon could be due to terminal boutons being slightly older than the rest of the arbor.

In summary, our data indicate that heterogeneity in release correlates highly with Ca^2+^ channel abundance and Ca^2+^ influx at AZs. Postsynaptically, PSDs apposed to high-releasing AZs display increased GluRIIA abundance and form segregated receptor fields, with GluRIIB forming a ring around a central core of GluRIIA. Release sites accumulate these high *P_r_* markers during a synapse maturation process in which newly formed AZs are consistently low *P_r_*, with AZs gaining signatures of high releasing sites over several days. Finally, mutations that increase or decrease presynaptic activity result in faster or slower rates of PSD maturation, respectively. These data add to our understanding of the molecular and developmental features associated with high versus low *P_r_* AZs.

## Materials and methods

### Drosophila stocks

Flies were cultured at 25°C on standard medium. Actively crawling third instar male and female larvae dwelling on top of the food were used for experiments unless otherwise noted. The following strains were used: UAS-myrGCaMP6s, UAS-GCaMP6m-BRP^short^, pBid-lexAop-myrGcaMP6s, UAS-myrjRGECO; Elav–GAL4, Mef2–GAL4, UAS-CacGFP (provided by Richard Ordway); UAS-CacTdTomato (provided by Richard Ordway); GluRIIA-RFP inserted onto chromosome III under the control of its endogenous promoter (provided by Stephan Sigrist), GluRIIB-GFP inserted onto chromosome III under the control of its endogenous promoter (provided by Stephan Sigrist); *nap^TS^* and *shaker^120b^, eag^1^* (provided by Barry Ganetzky); *BRP^69^* (provided by Stephan Sigrist); *BRP^def^; rab3^rup^* (provided by Ethan Graf) and 44H10-LexAp65 (provided by Gerald Rubin). *syt1* null mutants were generated by crossing *syt1^N13^*, an intragenic *syt1* deficiency (Littleton et al., 1994), with *syt1^AD4^*, which truncates Syt1 before the transmembrane domain (DiAntonio and Schwarz, 1994). *brp* null mutants were generated by crossing *brp^69^*, a truncation mutant, to a genomic deficiency *brp^def^.*

### Transgenic constructs

The fluorescent Ca^2+^ sensor GCaMP6s was tethered to the plasma membrane with an N-terminal myristoylation (myr) sequence. The first 90 amino acids of Src64b, containing a myristoylation target sequence, were subcloned into pBID-UASc with EcoRI and BglII (creating pBID-UASc-myr). GCaMP6s cDNA (Addgene plasmid 40753) was cloned into pBID-UASc-myr with BglII and XbaI. To generate the UAS-GCaMP6m-Brp-short line, GCaMP6m (Addgene plasmid 40754) cDNA and Brp-short (gift from Dr. Tobias Rasse) were PCR amplified and double digested with EcoRI/BglII and BglII/XbaI, respectively. The two cDNA fragments were ligated and the product was used to PCR amplify the fused GCaMP6m-Brp-short cDNA. The PCR product was inserted into the vector backbone pBID-UASc after digestion with EcoRI and XbaI to generate the final plasmid pBID-UASc-GCaMP6m-Brp-short. To create UAS-myrjRGECO, the vector backbone pBID-UASc-myr was digested with BglII and XbaI. jRGECO sequence was amplified from plasmid pGP-CMV-NES-jRGECO1a (gift from Dr. Douglas Kim, Addgene plasmid #61563). The digested backbone and insert were fused according to the Gibson assembly protocol using NEBuilder HighFidelity DNA Assembly Cloning Kit (E5520). To generate pBid-lexAop-myrGcaMP6s, myrGCaMP6s was amplified by PCR and inserted into pBiD-lexAop-DSCP (gift from Brian McCabe) between NotI and XbaI sites. All transgenic *Drosophila* strains were generated by BestGene.

### Immunocytochemistry

Wandering third instar larvae were dissected in Ca^2+^-free HL3 solution and fixed in 4% paraformaldehyde for 10 min, washed in PBT (PBS plus 0.1% Triton X-100) and blocked in 5% normal goat serum (NGS) and 5% BSA in PBT for 15 min. Samples were incubated overnight with anti-BRP (NC82, 1:200) from the Developmental Studies Hybridoma Bank (DSHB Cat# NC82, RRID:AB_2314866), washed for 1 hr in PBS and then incubated for 2–3 hr with Alexa Fluor 607-conjugated anti-mouse IgG at 1:1000 (Invitrogen, #A21237, RRID:AB_1500743).

### Confocal imaging and data analysis

Confocal images were obtained on a Zeiss Axio Imager two equipped with a spinning-disk confocal head (CSU-X1; Yokagawa) and ImagEM X2 EM-CCD camera (Hammamatsu). An Olympus LUMFL N 60X objective with a 1.10 NA was used to acquire GCaMP6s imaging data at 7 to 8 Hz. A Zeiss pan-APOCHROMAT 63X objective with 1.40 NA was used for imaging stained or live animals. third instar larvae were dissected in Ca^2+^-free HL3 containing 20 mM MgCl_2_. After dissection, preparations were maintained in HL3 with 20 mM MgCl_2_ and 1.3 mM Ca^2+^ for 5 min. To stimulate the NMJ, motor nerves were cut close to the ventral ganglion and sucked into a pipette. Single pulses of current were delivered every one second for myr-jRGECO mapping or every three seconds for GCaMP6s mapping with an AMPI Master-8 stimulator using a stimulus strength just above the threshold for evoking EJPs. A 3D image stack was taken before the GCaMP imaging session to generate a full map of GluRIIA or Cac channel distribution. Later, single focal planes were imaged continuously for 4–5 min to collect GCaMP signals. Volocity 3D Image Analysis software (PerkinElmer) was used to analyze images. All images were Gaussian filtered (fine) to reduce noise and a movement-correction algorithm was applied. To enhance identification of myrGCaMP6 flashes, background myrGCaMP fluorescence was subtracted by creating a composite stack of 5–6 images during intervals when no synaptic release occurred. To identify the position of GluRIIA receptors and corresponding Ca^2+^ events, a 3D stack image of GluRIIA was merged to create a single plane. AZ position was identified using the ‘find spot’ algorithm in Volocity 3.2 software that detects fluorescent peaks. ROIs with identical 5-pixel size (0.138 µm/pixel) were automatically generated by the software from identified GluRIIA spots. All GCaMP flashes were detected using the intensity threshold tool and assigned to specific ROIs based on proximity of their centroids. The time and location of Ca^2+^ events were imported into Excel or Matlab for further analysis. The number of observed GCaMP events per AZ was divided by the number of delivered stimuli to calculate AZ *P_r_*. Analysis of Cac, BRP, GluRIIA or GluRIIB intensities was performed similarly, identifying AZ fluorescence peaks and defining three pixel square ROIs around each peak to calculate average fluorescence. Average AZ fluorescence intensities of 3-pixel square ROIs was also used for correlation analysis.

### SIM and airyscan imaging

SIM microscopy was performed on an Applied Precision DeltaVision-OMX BLAZE-3D-Structural Illumination Microscope equipped with 60X, 1.4 NA oil objective and 3 sCMOS cameras. 3D-SIM images were obtained with 125 nm z-steps. Samples were illuminated by three central diffraction orders with 488, 562, and 640 nm lasers. For initial identification of specific NMJs, larvae were imaged in conventional confocal mode using a 20X oil objective. The positions of NMJ were marked and recorded to provide transition between objectives. A ZEISS LSM 800 microscope with Airyscan was also used to image anesthetized animals. Fluorescence was detected by a concentrically-arranged hexagonal detector array consisting of 32 single detector elements.

### Live imaging

Larvae were anesthetized with SUPRANE (desflurane, USP) from Amerinet Choice (Zhang et al., 2010). Larvae were incubated in a petri dish with a small paper towel containing Suprane for 1–2 min in a fume hood. Anesthetized larvae were positioned ventral side up on a glass slide between spacers made by transparent tape, which prevented extreme compression of the larvae. Different size spacers were required for the various larval stages. Larvae were covered with a thin film of halocarbon oil and then with a cover glass. NMJ synapses on muscle 26 in hemi-segment 2 or three were imaged. After an imaging session, larvae were placed in numbered chambers with food in a 25°C incubator. The same data acquisition settings where used to visualize NMJs at different larval stages. Larvae were imaged with either 6, 24 and 36 hr intervals for one data set (Figure 8A–C), or for 24 hr intervals for the remaining datasets. To keep the size consistent between different time periods, images of the corresponding NMJ area at younger stages were cut (dashed areas in figures) and placed onto a black background. This presentation generated a similar orientation of the different size NMJs for easier comparison for Figure 8, Figure 9 and Figure 8—figure supplement 1.

### Ionomycin application

Ionomycin (Sigma Aldrich) was dissolved in ethanol to make a 10 mM stock solution and was stored at 4°C. Ionomycin was used at a working concentration of 200 nM dissolved in HL3 with 1.3 mM Ca^2+^; this solution was applied to dissected preparations and NMJs were imaged one minute after application.

### Statistical analysis

Statistical analysis was performed with GraphPad Prism using one-way ANOVA followed by Dunnett’s Multiple Comparisons test for comparison of samples within an experimental group, or Student’s t-test for comparing two groups. Asterisks denote p values of: *p≤0.05; **p≤0.01; and ***p≤0.001. All histograms and measurements are shown as mean ± SEM. Pearson coefficient of correlation was calculated in GraphPad Prism using the following parameters: - two-tailed P value and 95% confidence interval.

## Additional files

Transparent reporting form

### Data availability

All data generated or analysed during this study are included in the manuscript and supporting files.
