# Supplementary material for: Characterization of developmental and molecular factors underlying release heterogeneity at Drosophila synapses
Source: eLife. 2018 Jul 10;7:e38268. doi: 10.7554/eLife.38268 (PMC6075867; doi:10.7554/eLife.38268)
Supplement: Peer review history

# Decision letter

**DOI:** 10.7554/eLife.38268.029

**Affiliations:** Baylor College of MedicineUnited States; University of Miami Miller School of MedicineUnited States; University of Southern CaliforniaUnited States

In the interests of transparency, eLife includes the editorial decision letter and accompanying author responses. A lightly edited version of the letter sent to the authors after peer review is shown, indicating the most substantive concerns; minor comments are not usually included.

Thank you for choosing to send your work, "Examining molecular determinants underlying heterogeneity of synaptic release probability using optical quantal imaging", for consideration at *eLife*. Your submission has been assessed by a Senior Editor in consultation with a member of the Board of Reviewing Editors (Hugo Bellen). Although the work is of interest, the editors felt more was needed to reveal enough new insight. However, they believe that if additional experiments are carried out and can answer specific questions, the manuscript could be suitable for *eLife* given the high quality of the data and the contribution to the field. This means that the work has potential. By rejecting the paper at this point you are free to move on to another journal at this time. On the other hand, if you feel the requested expeiments would substantially improve the manuscript and you wish to do these experiments (or have already done them), *eLife* would welcome a resubmission of this work.

If you decide to resubmit to *eLife*, some comments should be addressed in the submission. These particular comments are indicated in the reviews with quotation marks ("…").

Reviewer #1:

In this study, Akbergenova et al. seek to determine how the heterogeneity in release probability (Pr) is established across active zones at the *Drosophila* NMJ. Building from previous work this lab has pioneered in imaging presynaptic active zones (AZs) and the postsynaptic response from imaging single quantal release events, the authors now combine this optical quantal imaging with SIM microscopy and newly developed optical probes to provide compelling evidence for four main findings: First, *P_r_* is a stable feature of each active zone and is highly correlated with the intensity of the BRP scaffold and calcium channel Cac at AZs. Second, *P_r_* heterogeneity persists in Synaptotagmin mutants, consistent with AZ structures playing a primary role in determining *P_r_*heterogeneity. Third, the subset of high *P_r_*AZs acquire a change in the postsynaptic receptor field, including an enrichment in GluRIIA-containing receptors centrally opposed to AZs is observed, with GluRIIB-containing receptors located on the periphery. Finally, the authors undertake a challenging developmental analysis to provide evidence that the age of an active zone predicts *P_r_*, with the oldest AZs acquiring the most BRP/Cac material and hence exhibiting the highest *P_r_*.

Overall, this is a technically impressive study that reveals correlations between functional and structural features of AZs and PSDs at unprecedented resolution. The authors findings are consistent with work from many other labs identifying a positive correlation between increasing size, intensity, and/or abundance of AZ components with enhanced release probability. Further, the authors have provided very good evidence for biomarkers of high release probability at individual AZs that will be important for many researchers in the field, in which high *P_r_*AZs are defined by high intensities/size of BRP, Cac presynaptically and acquire the GluRIIA center/GluRIIB periphery structure. Although this central finding, that large active zones with high levels of calcium channels are highly correlated with high *P_r_*was known or strongly suggested from previous studies, the previous work did not put together functional and structural analyses together to demonstrate these properties at such high resolution. Further, it is particularly impressive that the authors go on to developmentally follow AZs as they mature, and provide evidence for what leads to AZs with high *P_r_*, which appears to be essentially the age of the AZ. These are all important contributions. However, I have three major criticisms on both technical and biological importance that need to be addressed. The authors frame this study as if there is an open question about whether AZs components are allocated heterogeneously and whether this has a significant impact on *P_r_*heterogeneity. It is not a major question or controversy that AZs with higher levels of calcium channels (and other associated machinery) will have a strong influence on *P_r_*. Studies at the Calyx of Held have demonstrated that calcium channels respond consistently with each action potential at active zones and obviously more channels will give more calcium influx, increasing release probability (see Sheng et al., 2012; Nakamura et al., 2015; Schneggenburger et al., 2012; Borst and Sakmann, 1996; Meinrenken et al., 2012; Sudhof, 2012, etc). Thus, “I think the authors should clearly lay this out in the Introduction and use their approach here to test the relative importance of AZ size/abundance vs other factors (synaptic vesicle proteins, modifications to AZ machinery) in determining *P_r_*heterogeneity”.

The technical rigor is what really stands out about this study, and though not particularly surprising, the validation of what many in the field have long suspected is still important. An important addition would be for the authors to estimate how much of the *P_r_*heterogeneity is conferred by size/abundance of BRP/Cac at AZs (see below).

Major points:

1) Framing of manuscript: The authors frame this study as if there was little evidence one way or the other to indicate the likely mechanisms that impose *P_r_*release heterogeneity at synapses. But as the authors point to towards the end the Discussion and in other areas of the manuscript, there is a large body of work that has already established that active zone size (and/or levels of AZ components at individual AZs) plays a major role in release probability and heterogeneity at synapses (see Holderith et al., 2012; Murthy et al., 2001; Matz et al., 2012; Ariel et al., 2012; Murthy et al., 1997; Rosenmund et al., 1993; Graf et al., 2009; Graf et al., 2012; Marrus and DiAntonio, 2004; Matkovic T et al., 2013). Further, it is well established that BRP size/intensity, as well as Cac intensity, varies considerably across AZs at the *Drosophila* NMJ; many studies have shown and quantified staining of BRP/Cac and other components at fly NMJs and shown a wide variation in size/intensity (Guerrero et al., 2005; Ehmann et al., 2014; Graf et. al, 2009; Paul et al., 2015; Peled and Isakoff, 2011). Several studies have also demonstrated that synaptic strength is positively correlated with such features (Wagh et al., 2006; Kittel et al., 2006; Kittel and Heckmann, 2016; Weyhersmuller et al., 2011). In addition to AZs, studies using Flash Photolysis at the Calyx of Held have shown that synaptic vesicles release with different intrinsic rates, independently of proximity to calcium sources (Wolfel et al., 2007; Schneggenburger et al., 2002). There is therefore a rich literature in which to frame the study as an investigation of the relative importance of AZ size/intensity/abundance vs other factors.

"Thus, the Introduction needs to be significantly revised to lay out from the outset what is known and suspected and give proper credit in setting up the motivation for this study."

I would strongly encourage and push the authors to frame the manuscript as an effort to define the relative contributions and importance of 1) AZ size/abundance of AZ material; 2) functional modifications (phosphorylation, etc); and 3) synaptic vesicle pools in determining the heterogeneity of *P_r_*at synapses. The authors find a strong correlation between BRP/Cac intensity and *P_r_*, which is not particularly surprising. But can the authors take advantage of their quantitative approach and seek to provide estimates of the relative importance of factors beyond AZ/Cac abundance in determining *P_r_*heterogeneity? Clearly there is not a perfect correlation with *P_r_*and Cac intensity, so can the authors say something about the importance of other factors in contributing to *P_r_*heterogeneity?

2) Investigate *P_r_*heterogeneity when AZ size/number is altered: The authors provide compelling evidence that *P_r_*heterogeneity persists in synaptotagmin mutants, indicating that heterogeneity is not absolutely dependent on a key determinant of *P_r_*from synaptic vesicles. In this case, is *P_r_*more strongly correlated with BRP/Cac abundance?

"It would be a great addition to show to what extent *P_r_*heterogeneity persists in a converse experiment – when AZ size is greatly enlarged (including BRP/Cac abundance), while AZ number/density is reduced in rab3 mutants (Graf et al., 2009). This would be of major interest and would nicely complement their study on syt mutants while serving to address the question on the relative importance of AZ size/abundance vs. synaptic vesicle/functional modifications in determining *P_r_*heterogeneity. "

3) Measuring calcium influx at AZs: The authors target the GCaMP6 reporter to active zones by fusing it to the BRPs sequence and claim that they are measuring differential calcium influx at each AZ (Figure 5 and 6). However, work from many groups have suggested that even the newest GCaMP reporters are too slow to capture the rapid calcium transients at AZ nanodomains, and instead the total calcium influx from all AZs equilibrates before GCaMP can capture the local change in concentration (see work from David DiGrigorio, Graeme Davis, Greg Macleod, etc). This is thought to be true for single action potential events and is even more of a concern using the 10Hz/5 sec paradigm by the authors. Hence, a very likely alternative explanation for the results shown in Figures 5 and 6 is that a similar concentration of calcium is experienced at each GCaMP-BRP puncta following the 10Hz stimulation, and that the abundance of the GCaMP reporter itself varies according to BRP/AZ size. Thus, the authors are simply reporting that GCaMP-BRP abundance correlates with BRP abundance at each AZ.

"The authors can easily show this by immunostaining their GCamp-BRP NMJ with anti-GFP and correlating the size/intensity with anti-BRP. "

Indeed, such as analysis was actually performed using a similar reagent published recently (BRPs-GCaMP6-mCherry; Kiragasi et al., 2017). The authors may wish to cite this study and/or even use this reagent to perform ratiometric calcium imaging with the mCherry tag, which may help to control for heterogeneity in abundance of the GCaMP reporter at AZs.

I don't think this is major point that detracts from the overall study, as higher BRP/Cac size/abundance almost certainly correlates with elevated calcium influx at individual active zones. But this section needs to be thoroughly revised to take into account this major possibility and the many caveats that the authors need to consider in their attempt to measure and interpret calcium influx at single active zones.

*Reviewer #2*:

This study used high resolution Calcium imaging to determine the components that contribute to or correlate with the heterogeneous distribution of AZ release probability *P_r_*. Although most of the results confirm what is already known about the AZ *P_r_*and therefore not surprising, providing direct measurement at such spatial and temporal resolution advances our understanding of the AZ *P_r_*. The first half of the paper showed that the *P_r_*heterogeneity is correlated with Calcium channel abundance and Ca^2+^ influx. The second half of the paper showed that the birth order of AZs determines the clustering of GluRII and *P_r_*. It remains unclear whether the accumulation of presynaptic AZ material is determined by birth order and whether the coupling of pre- and post-synaptic material accumulation during development requires neurotransmission.

1) Correlation between pre- and post-synaptic calcium events. In evoked events, presynaptic calcium influx at the AZ is required for synaptic vesicle fusion, and AZ calcium transients are necessary (but not sufficient) for EPSP, therefore, one would expect a stronger correlation between pre- and postsynaptic calcium events (determined in Figure 6) than that between calcium channel localization/abundance and postsynaptic calcium events (Figure 4). However, the authors observed almost opposite results where postsynaptic calcium events seem to correlate better with channel localization/abundance (r=0.62 Figure 4B) than with calcium influx (r=0.56, Figure 6B). The authors didn't offer any interpretation. The conclusion that "Ca^2+^ influx is the key factor that regulates evoked release at individual AZs" is not fully supported, as the results support a "correlation" between Ca^2+^ channel abundance/Ca^2+^ influx and *P_r_*. The actual "regulator", which determines the presynaptic Ca^2+^ channel abundance and local Ca^2+^ influx, still remains unknown. Also, in these experiments, it is unclear how the transgenes are expressed separately in the neuron and the muscle. The methods listed both transgenes, UAS-Cac-tdTomato, UAS-GCaMP, but no indication of GAL4 drivers used to express both either together or separately.

2) Developmental acquisition of *P_r_*property. The finding of developmental clustering of GluRII is fascinating. If the birth order of AZs determines the level of maturation and the accumulation of GluRII, then the birth order also determines *P_r_*and its heterogeneity.

"It needs to be determined whether the presynaptic terminal influences/contributes to the postsynaptic GluRII clustering. The authors indicated feasibility issues with *P_r_*map, however, measuring developmental/birth order clustering of Cac should be feasible, since Cac abundance is correlated with *P_r_*. In addition, the developmental clustering of Calcium channels and GluRII should be done in syt mutant background to determine the effect of transmission (evoked or spontaneous)."

3) NMJ specificity. Majority of the analysis focuses on the NMJ formed only by MN4-Ib onto muscle 4 at one time point (early L3 larvae). It will be valuable to at least include another NMJ, and include a different development stage (e.g., late L3 larve) for the *P_r_*map (like Figure 1A-C).

*Reviewer #3*:

The authors build on previous work performed by them and others to understand the variability in release probability (*P_r_*) seen at different active zones (AZ) at the *Drosophila* NMJ. They use some well-characterized and new genetic tools to measure calcium influx and localization of pre and post-synaptic machinery. Overall, they find Pr to be correlated with (a) BRP levels, (b) calcium channels (Cacophony), (c)Presynaptic calcium influx and d)GlurIIA levels at the PSD (Post synaptic density). They also determine that Synaptotagmin does not influence the heterogeneity of *P_r_*at different AZs. Finally, they do in-vivo imaging to determine that "birth order" of AZ seems to correlate with *P_r_*such that synapses that were formed earlier had higher *P_r_*. Technically, the experiments are done very well. As such, a very nice descriptive analysis of various aspects that control *P_r_*is presented.

The authors have failed to adequately test or describe the biological significance of the finding. Additionally, it is not clear how the findings presented significantly extend the body of knowledge that is present on how *P_r_*is set at the *Drosophila* NMJ or elsewhere. For instance, the variability of *P_r_*at different AZ at Muscle 4 has been demonstrated before and this has been correlated with levels of BRP (Peled, 2011). Levels of BRP is also correlated with the increased accumulation of Calcium channels (Fouquet, 2009) and this would presumably lead to higher calcium influx within these active zones. Furthermore, higher BRP levels are also correlated with higher GluRIIA levels at the PSD (Petzoldt, 2014).

If there is some finding that does diverge from previous work, the authors should make a stronger point to highlight that fact. Further genetic analysis could be used to delineate exactly how the different components (BRP, Cacophony, GlurII etc.) may affect *P_r_*and. whether these proteins play independent or dependent roles in controlling the heterogeneity of *P_r_*across AZ.

For example:

"The authors could investigate how the various BRP alleles affect the *P_r_*. The BRPnude allele does not seem to affect vesicle release but does affect synaptic depression. A conditional reversible knockdown of BRP has been documented (Nagarkar-Jaiswall et al., 2015). This allows one to control Brp levels."

"Does the reduction in Cacophony levels by RNAi or mutants change the heterogeneity of *P_r_*? would it be possible to use a calcium channel mutant with reduced activity to specifically differentiate whether the presence of the calcium channel is enough to define the high *P_r_*or the influx of calcium is needed?"

Similar analysis could be done with GluRII mutants or alleles such as GluRIIAE783A.

Furthermore, how does this developmentally controlled heterogeneity compare to synaptic plasticity? Are the identified high *P_r_*sites the most prone to synaptic depression? Are the low *P_r_*sites preferentially recruited to induce synaptic potentiation?

In the last section of the Results section, the authors correlate the number of mature AZs at the larval NMJ seen by in-vivo imaging with the number of high *P_r_*AZs observed in the previous *P_r_*mapping. They must avoid concluding that because both numbers are similar (14.7% and 10% respectively), it supports their hypothesis about the link between AZ maturation and high *P_r_.*These are very different experiments (in-vivo imaging vs larval preparations) and are also conducted at different NMJs (Muscle 4 vs Muscle 26). The authors should follow up their in vivo imaging with calcium imaging on larval preps to show that the mature AZ do in fact have high Pr.

# Reply: Author response

**DOI:** 10.7554/eLife.38268.030

We appreciate the reviewers’ comments on our manuscript and suggestions for improvement. All three reviewers were positive about the study and suggested several avenues for improving the presentation and extending the science, as well as refocusing how we presented the data. We have addressed their concerns in a point-by-point response below. In addition, we have added a host of new data and figures to extend our initial findings as suggested by the reviewers.

Reviewer 1 described the study as “a technically impressive study that reveals correlations between functional and structural features of AZs and PSDs at unprecedented resolution”. The reviewer requested three major clarifications and some additional minor comments we have now addressed as described below.

Framing of the manuscript: *“The Introduction needs to be significantly revised to lay out from the outset what is known and suspected and give proper credit in setting up the motivation for this study.”*

In our revised manuscript, we have focused our findings in the context of what is known in the field as suggested by the reviewer. We indicate the well-described previous links in the field to Ca^2+^ channels and Ca^2+^ influx in relation to synapse strength. We have reframed our data presentation as the reviewer suggests to lay out the relative importance of the AZ components versus other factors. In addition, we have shifted the last half of the study to investigate how activity alters PSD maturation during development to provide new insights that further separate our study from prior work.

Investigate P_r_ heterogeneity when AZ size/number is altered: "*It would be a great addition to show to what extent P_r_ heterogeneity persists in a converse experiment – when AZ size is greatly enlarged (including BRP/Cac abundance), while AZ number/density is reduced in rab3 mutants (Graf et al., 2009). This would be of major interest and would nicely complement their study on syt mutants while serving to address the question on the relative importance of AZ size/abundance vs. synaptic vesicle/functional modifications in determining P_r_ heterogeneity. "*

As requested by the reviewer, we completed a new set of experiments in the *rab3* mutant and added new data and figures on this topic. Prior work with *rab3* (Peled and Isacoff, 2011) had been done with optical quantal imaging to examine *Pr* in the mutant, revealing that the majority of sites have low *Pr*, with a smaller population of large release sites with much higher *Pr*. We observed a similar phenotype in our study and were able to extend the analysis by using the redistribution of *Pr* seen in the *rab3* NMJ as atool to examine the activity-dependence of postsynaptic glutamate receptor field maturation on presynaptic activity using intravital imaging across development (new Figure 10 and Figure 10—figure supplement 1). While prior studies of *rab3* mutants examined the third instar stage, we used sequential intravital imaging throughout the whole developmental window beginning in first instar larvae to determine how the NMJ develops with only a few large AZs containing Cac and BRP, while the rest of the AZs lack these components. We observed that glutamate receptor fields apposing BRP-enriched sites developed into a more mature state than PSDs apposing sites lacking BRP and Cac. This observation suggests that PSD maturation depends on either presynaptic components or presynaptic release at the level of single release sites. Coupled with new data we provide on other mutants that enhance or decrease neuronal activity, our findings indicate that the level of synaptic activity at single AZs is a key factor in controlling the rate of postsynaptic maturation of the glutamate receptor field.

Measuring calcium influx at AZs: *“A very likely alternative explanation for the results shown in Figures 5 and 6 is that a similar concentration of calcium is experienced at each GCaMP-BRP puncta following the 10Hz stimulation, and that the abundance of the GCaMP reporter itself varies according to BRP/AZ size. I don't think this is major point that detracts from the overall study, as higher BRP/Cac size/abundance almost certainly correlates with elevated calcium influx at individual active zones. But this section needs to be thoroughly revised to take into account this major possibility and the many caveats that the authors need to consider in their attempt to measure and interpret calcium influx at single active zones.”*

As the reviewer correctly points out, differences in BRP levels at individual AZs may affect our measurement. Although the use of ratiometric GCaMP-mcherry sensors for this work would be great, it is not feasible for our *Pr* mapping as we need the red channel for postsynaptic Ca^2+^ measurements to assign synaptic vesicle fusion events to individual AZs. As such, we have reworked this section to highlight this important caveat noted by the reviewer. We note that the GCaMP-BRP sensor data is used to support our conclusions drawn from the Cac localization studies and *Pr* mapping.

In addition, we experimentally addressed the reviewers comment as well. The nc82 antibody recognizes an epitope at the C-terminus of BRP and thus also recognizes BRP^short^. Therefore, we were unable to stain for endogenous BRP in this line without also labeling GCaMP-BRP^short^. We therefore approached this question using a different method; we applied the Ca^2+^ ionophore ionomycin to uniformly elevate intracellular Ca^2+^ throughout the presynaptic terminal. We then measured the heterogeneity in fluorescence from GCaMP-BRP^short^ before applying ionomycin (baseline fluorescence), during 10 Hz stimulation, and after ionomycin application. In the presence of ionomycin, differences in fluorescence signals between AZs should be entirely due to heterogeneity in sensor abundance. We observed a rightward shift in the GCaMP-BRP^short^ intensity distribution among AZs upon ionomycin application compared to 10 Hz stimulation (Figure 5—figure supplement 1A, B), indicating that detection of Ca^2+^ by GCaMP-BRP^short^ fluorescence during 10 Hz stimulation is not limited by sensor abundance. Furthermore, we observed a significant difference in the shape of the distribution during 10 Hz stimulation compared to both before stimulation and after ionomycin. The distribution of fluorescence intensities is narrower both at rest and upon ionomycin application; these two distributions should primarily reflect sensor distribution. In contrast, the distribution of GCaMPBRP^short^ fluorescence upon 10 Hz stimulation is wider, indicating that the sensor is reporting local changes in Ca^2+^ influx and not just sensor distribution (Figure 5—figure supplement 1C, D). Thus, although GCaMP-BRP^short^ abundance is likely to contribute to the levels of Ca^2+^ influx detected, these results are consistent with heterogeneity in Ca^2+^ influx across individual AZs. We have modified the text to clearly explain this caveat and have added a new supplemental figure for the ionomycin experiment (Figure 5—figure supplement 1).

We also added another experimental approach to further strengthen the connection between *Pr* and Ca^2+^ influx. We reasoned that if Ca^2+^ influx at AZs, and not just Cac abundance, is a driving factor in release heterogeneity, then specifically reducing the conductance of Cac should reduce *Pr* across the entire range of release, effectively shifting the distribution towards lower *Pr* but maintaining the overall shape of the distribution. As such, we performed *Pr* mapping in the *cac^NT27^* mutant, which has a point mutation in the S4 voltage sensor that reduces Ca^2+^ influx and observed the expected change. These data provide functional validation that Ca^2+^ levels are important for overall *Pr* and are not simply correlated (not that this is a controversial assertion given the large number of studies demonstrating this point).

Reviewer 2 found the developmental analysis fascinating and suggested some valuable additional experiments and some clarifying modifications to the text.

1a) Correlation between pre- and post-synaptic calcium events. *In evoked events, presynaptic calcium influx at the AZ is required for synaptic vesicle fusion, and AZ calcium transients are necessary (but not sufficient) for EPSP, therefore, one would expect a stronger correlation between pre- and postsynaptic calcium events (determined in Figure 6) than that between calcium channel localization/abundance and postsynaptic calcium events (Figure 4). However, the authors observed almost opposite results where postsynaptic calcium events seem to correlate better with channel localization/abundance (r=0.62 Figure 4B) than with calcium influx (r=0.56, Figure 6B). The authors didn't offer any interpretation.*

The results represent correlations over a very large number of AZs across multiple animals. Although it is unclear why the correlation with Ca^2+^ influx is slightly lower than with Ca^2+^ channel density, it may be reflective of the fact that we have to measure Ca^2+^ influx during a short train of action potentials rather than with the single stimuli we use to measure *Pr*. As such, the Ca^2+^ influx measurements are not direct assays for single action potential Ca^2+^ transients, and certainly reflect summation of signals over the short train. However, they do support the model that AZs with higher Ca^2+^ channel density and overall higher Ca^2+^ influx are likely to be higher *Pr* synapses. As described in reviewer 1 comment 3, we also added new functional data on the effect of a *Cac* mutant and its ability to support release as a mechanism to strengthen the model.

1b) Clarifying correlation vs. causation in release*. The conclusion that "Ca^2+^ influx is the key factor that regulates evoked release at individual AZs" is not fully supported, as the results support a "correlation" between Ca^2+^ channel abundance/Ca^2+^ influx and P_r_.*

We agree with the reviewer that causation cannot be inferred directly from the correlation between Cac abundance/Ca^2+^ influx and *Pr*. For example, Ca^2+^ influx could vary between AZs without serving as the limiting reagent for vesicle fusion. In response to this concern, we performed an additional genetic manipulation to test whether Ca^2+^ influx through presynaptic Ca^2+^ channels, independent of the structural presence of the channel, is truly a causative factor in evoked release at *Drosophila* NMJs. When we mapped release probability in *cac^NT27^* mutants, which have lower conductance due to a point mutation in the S4 voltage sensor, we observed a significant downward shift in the distribution of *Pr* at the NMJ (Figure 6—figure supplement 1). This suggests that amount of Ca^2+^ influx through Ca^2+^ channels, rather than the mere presence of the channels, directly effects release at AZs across the release spectrum.

1c) The regulator of presynaptic calcium channel abundance. *The actual "regulator", which determines the presynaptic Ca^2+^ channel abundance and local Ca^2+^ influx, still remains unknown.*

Through our developmental analysis of AZ maturation using intravital imaging, we have shown that one key primary regulator of presynaptic Ca^2+^ channel abundance is age. Ca^2+^ channels accumulate at AZs throughout their maturation process. We demonstrated that AZs add Cac throughout their development by following Cac-GFP and GluRIIA-RFP over time. Unlike the Cac-GFP area, the GluRIIA-RFP field is large enough to be reliably resolved by light microscopy, and we observed that the size and intensity of the GluRIIA field increases over time as AZs mature. Moreover, we saw a strong correlation between Cac-GFP intensity and GluRIIA intensity, suggesting these two components are tightly coupled during synapse maturation. Based on this information, we suggest that Cac abundance at AZs increases during maturation and that AZ age is a major factor in determining AZ Ca^2+^ channel abundance. There are likely to be many interesting mechanisms further regulating the rate of Ca^2+^ channel accumulation and persistence at AZs, as well as the regulation of Ca^2+^ channel gating at individual AZs, and these are topics of interest moving forward.

1d) Clarifying expression of transgenes. *“It is unclear how the transgenes are expressed separately in the neuron and the muscle. The methods listed both transgenes, UAS-CactdTomato, UAS-GCaMP, but no indication of GAL4 drivers used to express both either together or separately.”*

We thank this reviewer for pointing out the lack of clarity in our methods of transgene expression. In this study, all presynaptically driven transgenes (UAS-Cac-tdTomato, UAS-CacGFP, and UAS-GCaMP-BRP^short^) were expressed using the pan-neuronal driver elav-GAL4. Postsynaptically, UAS-myr-GCaMP6s was driven with the muscle driver *mef2*-GAL4 when coexpressed with GluRIIA-RFP, which was inserted on chromosome III and driven using its endogenous promoter. Myr-GCamP6s was driven using the LexA/LexOP system in muscle when combined with GluRIIA-RFP and GluRIIB-GFP which were both inserted on chromosome III and driven using their endogenous promoters. This has been clarified throughout the text.

Developmental acquisition of P_r_ property. The finding of developmental clustering of GluRII is fascinating. If the birth order of AZs determines the level of maturation and the accumulation of GluRII, then the birth order also determines P_r_ and its heterogeneity… It needs to be determined whether the presynaptic terminal influences/contributes to the postsynaptic GluRII clustering. The authors indicated feasibility issues with P_r_ map, however, measuring developmental/birth order clustering of Cac should be feasible, since Cac abundance is correlated with P_r_. In addition, the developmental clustering of Calcium channels and GluRII should be done in syt mutant background to determine the effect of transmission (evoked or spontaneous)."

This was an excellent set of suggestions, and we performed an extensive number of experiments to address these questions; this new addition enhanced the novelty of this work and we are grateful to the reviewer for contributing these ideas. We addressed the question of whether differences in presynaptic activity levels can alter the maturation rate of synapses at the level of individual AZs. Instead of monitoring Cac-GFP and GluRIIA-RFP over time like the reviewer suggested, we chose to perform these experiments using GluRIIA-RFP and GluRIIB-GFP for two reasons. First, GluRIIA and GluRIIB co-labeling allows us to identify PSDs that display receptor subtype segregation, which is a useful marker for the progression of PSDs through maturation. Second, Cac-GFP puncta are smaller than the resolution limit of conventional light microscopy, and so all quantification through the cuticle would rely on comparisons in Cac puncta intensity that could be affected by bleaching and by varying cuticle thickness.

We began by measuring the rate of PSD growth (24-hour fold-increase in GluRIIB area) and maturation (percent of PSDs displaying GluRIIA/GluRIIB segregation) in *BRP^69/def^* mutants that (1) alter presynaptic structure and (2) decrease release by reducing Cac abundance at AZs. In this mutant we observed a significant reduction in both PSD growth and ring formation. In *syt1^null^,* and *nap^TS^* mutants that reduce presynaptic release without structural changes to the AZ, we also observed a significant reduction in postsynaptic maturation rate (Figure 10). In the *shaker, eag* double mutant with increased presynaptic excitability, we observed a significantly increased rate of GluRIIB field size increase when compared to control, and a significant increase in GluRIIB rings at the early second instar stage (Figure 10). Finally, we used the *rab3* null mutant to investigate whether differences in PSD growth and maturation rate could occur between AZs containing BRP and Cac versus neighboring AZs within the same NMJ that are deficient in these components. In the first instar stage when AZs are roughly age matched, we saw that release sites enriched in presynaptic components had developed large and mature PSDs in stark comparison with un-enriched AZs, whose PSDs were underdeveloped (Figure 10, supplementary figure 1). Overall, these results strongly suggest that presynaptic activity drives postsynaptic maturation rate at an AZ-specific level and opens many exciting questions for future work.

NMJ specificity. Majority of the analysis focuses on the NMJ formed only by MN4-Ib onto muscle 4 at one time point (early L3 larvae). It will be valuable to at least include another NMJ, and include a different development stage (eg, late L3 larve) for the P_r_ map (like Figure 1A-C).

We agree that *Pr*mapping at a different developmental stage and another NMJ are valuable additions to this study, even beyond their ability to determine how generalizable our observations of release heterogeneity are across synapses and across developmental stages. Mapping release at muscle 26 serves to connect the work we have done building correlations between release and synapse component density in muscle 4 with the work performed following AZ maturation over development at muscle 26. We were pleased to see that when we mapped release at muscle 26 in the third instar stage, the distribution in *Pr*was indistinguishable from the distribution seen at the NMJ on muscle 4 (Figure 9—figure supplement 1). This suggests that our findings in these two NMJs are indeed comparable, and that *Pr* heterogeneity is not specific to muscle 4. Mapping release in the second instar provides another way to test our model that AZ age and developmental maturation are key determinants of *Pr* heterogeneity during NMJ development. Based on our observation that the number of AZs roughly doubles every 24 hours of development, and that AZs are born weak and undergo a multi-day maturation process where they develop high *Pr*, we would expect the second instar NMJ to show a shift in *Pr* distribution when compared to the third instar NMJ. Specifically, we would expect a lower percentage of low *Pr* sites in second instar stage (as the number of AZs will double over the next 24 hours and the newly added AZs will be low *Pr*). Indeed, when we mapped *Pr* in the second instar stage, we observed the expected shift in the distribution ((Figure 9—figure supplement 1)), providing support for the model that developmental maturation and new AZ addition over time generates heterogeneity at the NMJ.

Reviewer 3 indicated the manuscript was technically done very well but wanted clarification on the biological significance of the findings and the novelty of the current data.

1a) Describing biological significance.

We thank this reviewer for pointing out the lack of discussion on the novelty of this work, and we have altered the text significantly to further contextualize these results. Though it has already been shown that *Pr* heterogeneity exists across the NMJ (Peled 2011, Melom 2013) and that *Pr* correlates with levels of BRP (Reddy-Alla et al., 2017, Muhammad et al., 2015, Peled et al., 2014), the work presented here extends this body of knowledge in multiple important ways.

Combining our green Ca^2+^ sensor with GluRIIA-RFP to mark individual release sites has allowed us to provide a higher resolution view of the correlation between single AZ evoked release and BRP, Cac, and GluRIIA abundance. GluRIIA-RFP also allowed us to employ superresolution structured illumination microscopy after quantal imaging to confirm that high releasing sites correspond to single AZs. This increase in resolution is essential for understanding release heterogeneity at the NMJ, where single BRP T-bars are smaller than the resolution of conventional light microscopy and can be closely clustered.

We have assessed the role of local synaptic vesicle pools in determining local release heterogeneity. Though it has been established that certain synaptic vesicle components (like Syt1) are dominant factors in determining *Pr* at synapses, the role of local synaptic vesicle pools in determining release heterogeneity across AZs of the NMJ has not been explored. By showing that heterogeneity in release is retained in *syt1* null mutants, and is stable during extensive synaptic vesicle cycling, the study indicates that AZ-specific, differentially fusogenic synaptic vesicle populations are unlikely to underlie the heterogeneity in release observed at the NMJ.

We have shown that AZ maturation throughout the three stages of larval development plays a dominant role in generating the extreme release heterogeneity that we and other labs have observed in the third instar larval stage. The Sigrist lab has previously demonstrated that AZs gain presynaptic and postsynaptic components over time, generating the hypothesis that AZ aging process may lead to increasing AZ *Pr*. However, to demonstrate that the heterogeneity observed at the third instar stage results largely from AZ birth date, we needed to extend this intravital imaging of individual release sites through a longer time period starting much earlier in development to show that the heterogeneity in developmental birth order is a major source of heterogeneity in AZ release probability at the third instar stage. This long-term developmental perspective, extending from early first to third instar stages, provides an important developmental picture of how release heterogeneity arises. Since the field typically uses the third instar NMJ as a model to study presynaptic release, understanding how functional release heterogeneity seen at the third instar NMJ is established from a molecular and developmental perspective provides valuable information about this common model synapse.

We have included additional experiments to show that postsynaptic maturation rate is activity-dependent and can occur at an active zone-specific level (this is discussed below). To our knowledge, this has not been previously demonstrated at the NMJ. Overall, these results provide a more developmentally complete and technically higher resolution understanding of how release heterogeneity at the NMJ arises during development.

*1b)* Furthering biological significance through more genetic analysis. *“Further genetic analysis could be used to delineate exactly how the different components (BRP, Cacophony, GlurII etc.) may affect P_r_ and. whether these proteins play independent or dependent roles in controlling the heterogeneity of P_r_ across AZ… The authors could investigate how the various BRP alleles affect the P_r_.The BRPnude allele does not seem to affect vesicle release but does affect synaptic depression. A conditional reversible knockdown of BRP has been documented (Nagarkar-Jaiswall et al., 2015). This allows one to control Brp levels."*

This reviewer encouraged us to move beyond building correlations between AZ components and release probability in wildtype animals and to begin using genetic manipulations to further understand the generation of heterogeneous *Pr* at the NMJ. Since we have shown that AZ age is a strong contributor to the abundance of pre- and postsynaptic proteins that our lab and others have shown to correlate with *Pr* heterogeneity at the NMJ, we added an entire set of new experiments to investigate what parameters affect age-dependency of *Pr*. First, we established confidence in the age-dependency of *Pr* using two new experiments; we showed that all new active zones (under 24 hours old) are low *Pr* (Figure 9F, G). Second, we mapped *Pr* in the second instar stage and showed that, in line with what our model predicts, the heterogeneity in release in the second instar is shifted towards higher releasing sites, with a reduction in low-releasing sites (Figure 9—figure supplement 1).

After further documenting the age-dependence of *Pr*, we addressed the concern that further genetic manipulations could be used to determine how overall component accumulation during development could shape *Pr* distribution by assaying whether mutants that alter presynaptic activity can influence synapse maturation rate (Figure 10). We measured the rate of PSD growth (24-hour fold-increase in GluRIIB area) and GluRIIA/B segregation in mutants with altered presynaptic activity. In the *BRP^69/def^, syt1^null^,* and *nap^TS^* mutants with decreased presynaptic release, we observed a significant reduction in postsynaptic maturation rate and a significant reduction in GluRIIA/IIB rings (Figure 10). In the *shaker, eag* double mutant with increased presynaptic excitability, we observed a significantly increased rate of GluRIIB field size increase compared to control, and a significant increase in GluRIIB rings at the early second instar stage (Figure 10). Finally, we used the *rab3* null mutant to investigate whether differences in PSD growth and maturation rate could be seen between AZs containing BRP and Cac vs. neighboring AZs within the same NMJ that are deficient in these components. In the first instar stage when AZs are roughly age matched, we observed that release sites enriched in presynaptic components had developed large and mature PSDs in stark comparison with un-enriched AZs, whose PSDs were underdeveloped (Figure 10—figure supplement 1).

Overall, these results add valuable new information to the study; we have shown that not only is the heterogeneity in AZ *Pr* explained by differences in AZ component density that reflect differences in AZ age, but we have also shown that this dependence on age can be influenced by changes in presynaptic activity.

*1c)* Functional demonstration that calcium influx determines P_r_. *“Does the reduction in Cacophony levels by RNAi or mutants change the heterogeneity of P_r_? would it be possible to use a calcium channel mutant with reduced activity to specifically differentiate whether the presence of the calcium channel is enough to define the high P_r_ or the influx of calcium is needed?"*

This is an excellent suggestion. As another approach to test whether the level of Ca^2+^ influx rather than the structural presence of the Ca^2+^ channel is responsible for determining *Pr*, we generated *Pr* maps in the *cac^NT27^* mutant using dual color quantal imaging, with GluRIIA-RFP and myrGCamP6s expressed postsynaptically in the muscle. Cac^NT27^ channels have reduced Ca^2+^ conductance due to a point mutation in the Cac S4 voltage sensor (Rieckhof et al. 2003). We observed that *cac^NT27^* results in a global decrease in *Pr* across AZs; WT evoked *Pr* ranged from 0 to 0.73 with an average of 0.073, while *Cac^NT2^ Pr* ranged from 0 to 0.47 with a significantly lower average of 0.049 (Figure 6—figure supplement 1). In this mutant, the entire *Pr* distribution was shifted towards low *Pr* when compared to controls, and the resulting NMJ was almost entirely populated with low-releasing AZs. This result strongly suggests that the levels of Ca^2+^ influx through Cac channels, and not the physical presence of the channels, is a primary determinant of *Pr* at AZs.

*1d)* Differential short term plasticity across AZs *“Furthermore, How does this developmentally controlled heterogeneity compare to synaptic plasticity? Are the identified high P_r_ sites the most prone to synaptic depression? Are the low P_r_ sites preferentially recruited to induce synaptic potentiation?”*

This is a compelling question, and the *Drosophila* NMJ provides an excellent model system to dissect the short-term plasticity at low vs. high *Pr* sites. These questions have been previously addressed by Peled and Isacoff (2011); here, the authors demonstrated that high releasing AZs tend to depress during paired pulse stimulation, while lower releasing AZs facilitate. Thus, the developmentally generated heterogeneity in *Pr* across AZs results in a population of AZs with distinct short-term plasticity properties. We have included a description of this result in the text.

1e) In the last section of the Results section, the authors correlate the number of mature AZs at the larval NMJ seen by in-vivo imaging with the number of high P_r_ AZs observed in the previous P_r_ mapping. They must avoid concluding that because both numbers are similar (14.7% and 10% respectively), it supports their hypothesis about the link between AZ maturation and high P_r_. These are very different experiments (in-vivo imaging vs. larval preparations) and are also conducted at different NMJs (Muscle 4 vs. Muscle 26). The authors should follow up their in-vivo imaging with calcium imaging on larval preps to show that the mature AZ do in fact have high P_r_.

This is an excellent suggestion, and we have altered the text and performed two additional experiments to address this point. First, as the reviewer pointed out, it is not ideal to compare release distributions at muscle 4 versus muscle 26 without directly demonstrating that these two NMJs have a similar distribution of *Pr*. To address this issue, we mapped release at muscle 26 using the same methods that were used to map release in our initial experiments at muscle 4. When we compared the release distributions at the two NMJs, we saw that the distributions were not significantly different (Figure 9—figure supplement 1). Second, we followed the suggestion of this reviewer to directly assess whether AZ age corresponds to *Pr* by mapping *Pr* after determining the developmental age of AZs using intravital imaging. We followed PSDs in animals expressing GluRIIA-RFP, GluRIIB-GFP, and myr-GCaMP for 24 hours using in vivo imaging, and then dissected the animal and mapped *Pr* (Figure 9F). We observed that PSDs that appeared on the second day of imaging (less than 24 hours old) were consistently associated with very low-*Pr* AZs. The mean *Pr*of newly-formed AZs was 0.035, and the *Pr* for AZs older than 24 hours was 0.15 (Figure 9G). This result supports a model in which a vast majority of AZs are born weak, and higher Pr takes more than 24 hours to develop.
